# Linking host morphology and symbiont performance in octocorals

**DOI:** 10.1038/s41598-018-31262-3

**Published:** 2018-08-27

**Authors:** Sergio Rossi, Nadine Schubert, Darren Brown, Marcelo de Oliveira Soares, Victoria Grosso, Emma Rangel-Huerta, Ernesto Maldonado

**Affiliations:** 1 The Environmental Science and Technology Institute, Autonomous University of Barcelona, Campus UAB s/n, Barcelona, 08193 Spain; 2 Unidad Académica de Sistemas Arrecifales Puerto Morelos, Instituto de Ciencias del Mar y Limnología, Universidad Nacional Autónoma de México, Puerto Morelos, 77580 Mexico; 3 Department of Biology, The Pennsylvania State University, University Park, Pennsylvania 16802 USA; 4 Instituto de Ciências do Mar (Labomar), Universidade Federal do Ceará, Fortaleza, 60165-081 Brazil; 5 Present Address: DiSTeBA, University of Salento, Lecce, 73100 Italy; 6 Present Address: Programa de Pós-graduação em Oceanografia, Centro de Ciências Físicas e Matemáticas, Universidade Federal de Santa Catarina, Campus Trindade, Florianopolis, 88040-970 Brazil

## Abstract

Octocorals represent an important group in reef communities throughout the tropical seas and, like scleractinian corals, they can be found in symbiosis with the dinoflagellate *Symbiodinium*. However, while there is extensive research on this symbiosis and its benefits in scleractinians, research on octocorals has focused so far mainly on the host without addressing their symbiosis. Here, we characterized and compared the photophysiological features of nine Caribbean octocoral species with different colony morphologies (sea fan, plumes, whips and rods) and related key morphological features with their respective symbiont photobiology. Colony features (branch shape and thickness), as well as micromorphological features (polyp size, density), were found to be significantly correlated with symbiont performance. Sea fans and plumes, with thinner branches and smaller polyps, favor higher metabolic rates, compared to sea rods with thicker branches and larger polyps. Daily integrated photosynthesis to respiration ratios > 1 indicated that the autotrophic contribution to organisms’ energy demands was important in all species, but especially in sea whips. This information represents an important step towards a better understanding of octocoral physiology and its relationship to host morphology, and might also explain to some extent species distribution and susceptibility to environmental stress.

## Introduction

In coral reefs, hexacorals, octocorals and sponges are the most important contributors to the living three-dimensional structures that give complexity to the system. Their diversity and complexity contribute to the correct functioning of the biogeochemical cycles, as well as to the productivity and nursing effect of these complex structures^1^. Octocorallia constitute, among these sessile suspension feeders, a group with significant presence in shallow tropical reefs, where they represent important structural components of the community^2,3^. In these environments, a large proportion of the octocoral species, as with many other reef organisms, are well known for engaging in photosymbiosis with unicellular algae, which provides them with metabolic advantages in nutrition^4^. So far, most of the photobiological approaches and the analysis of the predicted impacts of global change on species fitness in coral reefs have been performed on scleractinians. Octocorals, in this complex panorama, have been so far largely neglected^4^.

The most widespread coral reef photosymbionts contributing to the energy budget of mixotrophic cnidarians are dinoflagellates of the genus *Symbiodinium*. In scleractinians it has been shown that the symbiont cells produce photosynthates, organic compounds like glycerol and triglycerides that are translocated within and between cells to supplement the host nutrient requirements (up to 95% of that required by the host, e.g.^5^). The importance of the heterotrophic and autotrophic input in these suspension feeders varies between species of a given taxonomic group. Even though in some corals most of their energetic demands can be fulfilled through the symbiosis, they still depend partially on heterotrophic feeding (mixotrophy)^6^. Among the most important features to be considered in the optimization of light resources are certain morphological traits^7^ that may be essential to understand the dependence on autotrophic and heterotrophic resources. Porter^8^ showed that in scleractinian corals surface area to volume ratio (SA/V) and polyp size might be good indicators for the importance of light and zooplankton capture, respectively. Maximum SA/V ratios result from branching or plating morphologies and are optimally suited for light capture. This feature is accompanied by small polyp size^8^. Species with low SA/V usually have larger polyps, which are potentially better suited for zooplankton capture. Thus, Porter^8^ suggested that species with high SA/V present higher photosynthesis to respiration ratios (P/R), which is often used as an indicator of the autotrophic contribution to cnidarian metabolism^4^.

Both overall colony morphology as well as micromorphology of skeletal elements (sclerite) traditionally had an important role in the taxonomic classification of soft corals^9^. Octocorals have a great variety of colony shapes, from flattened and branching fan-like to whip-like, feathery, and rod-like forms, accompanied by highly variable polyp sizes^10,11^. Morphology has various roles in the biological functioning of cnidarian species^12^, but some of them that may be essential in understanding the autecology remain largely unexplored. Thus, according to the conclusions drawn by Porter^8^ for scleractinians, branch morphology and polyp size in octocorals might be features indicating high or low autotrophic input, as it directly affects the SA/V. Branched morphologies favor higher SA/V, while smaller polyps ensure higher light absorption for the symbionts. Thus, it could be inferred that species with those features might be relying more on autotrophic input compared to others.

So far, few attempts have been made to compare the photophysiological performance of different octocoral species^13,14^ and only one study has attempted to relate the potential autotrophic contribution, using P/R as proxy, to the species’ morphological traits^15^. The results of that study agree with Porters’ conclusions, showing a correlation between species’ colony morphology and polyp size and their trophic strategy. Thinner-branched species with small polyps were found to be net autotrophic (P/R>1.5), in contrast to thicker-branched species with larger polyps.

Based on the scarce information available, an in-depth approach evaluating the photosynthetic performance of octocorals in relation to their micro- and macro-morphological traits will improve our understanding of species’ reliance on autotrophic energy input, and hence, help explain their depth distribution and possible differences in their sensitivity to environmental changes. The latter point is specifically important in view of the threats currently imposed on coral reefs by stressors related to global change (i.e., ocean acidification, warming) and to local perturbations (e.g. eutrophication), as it might be hypothesized that species with a higher dependence on autotrophic contribution might be at a disadvantage during thermal stress events due to the need of higher energy investments for antioxidant defenses to counteract the formation of reactive oxygen species.

In this study, the photophysiological characteristics of nine representative Caribbean octocoral species were determined and compared, and their relationship with the species morphological traits were explored. To achieve this objective, certain morphological features (branch thickness, SA/V, polyp size, sclerite size-abundance-color, *Symbiodinium* distribution in the polyp and connective coenenchyma) of nine species, separated into different groups based on the aforementioned features, were related to the species’ photosynthetic characteristics (gross photosynthesis, photosynthetic efficiency, dark respiration, P/R, *Symbiodinium* cell density, pigment content). For comparative purposes with other studies, the photosynthetic variables were normalized to different parameters. However, our results and discussion focus on parameters normalized to symbiont cell density and ash-free dry weight, as the main goal was to explore the relationships between species morphology and symbiont performance and productivity. Finally, we evaluated the importance of autotrophic (P/R) and heterotrophic input (δ^13^C, δ^15^N) to the energy budget of the different species.

## Results

### Comparison of species’ morphological traits

**Figure 1:**
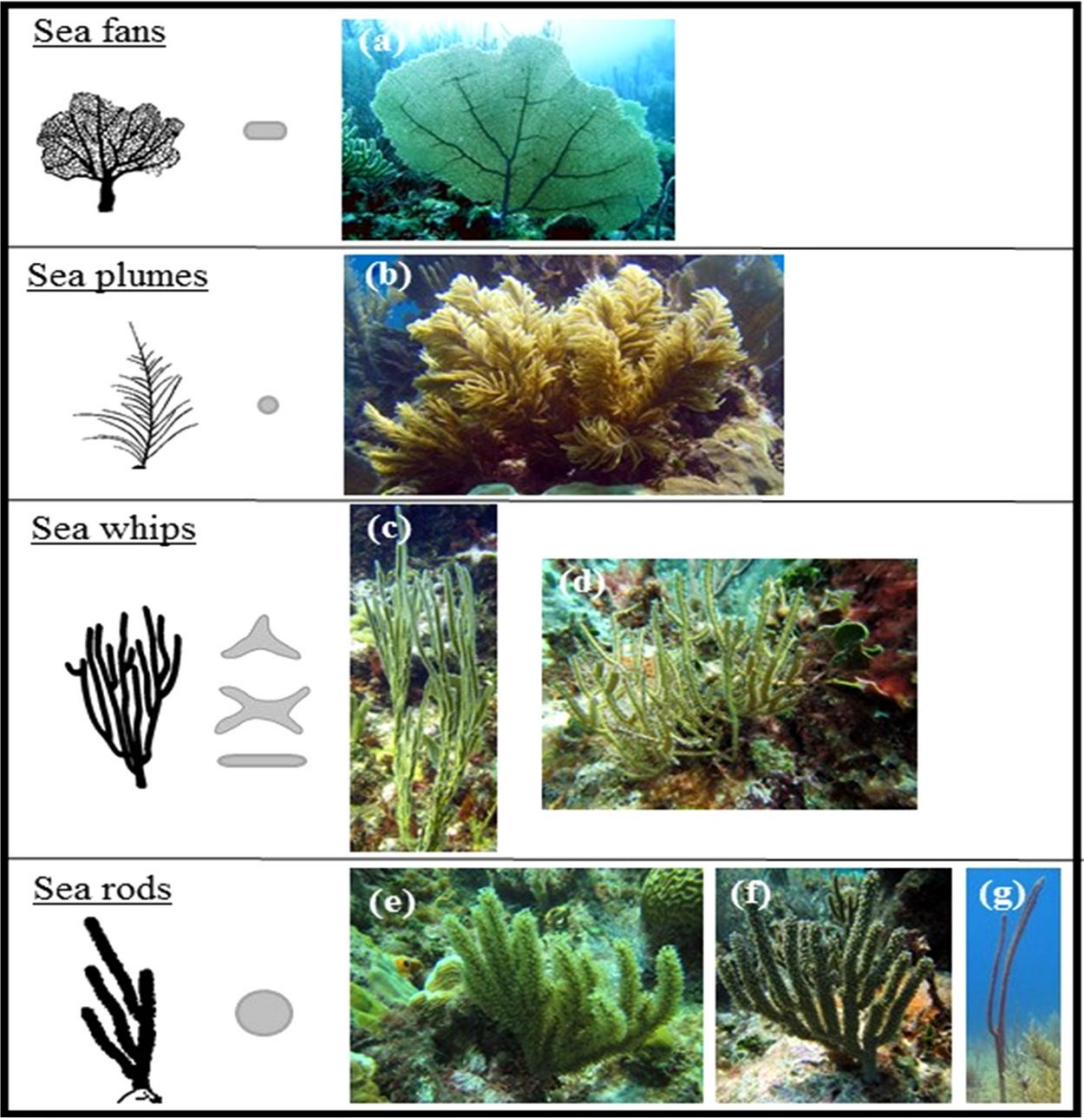
Photos of the studied Caribbean octocoral species, showing their differences in branch morphologies: (**a**) *Gorgonia ventalina*, (**b**) *Antillogorgia americana*, (**c**) *Pterogorgia anceps*, (**d**) *Pterogorgia citrina*, (**e**) *Eunicea mammosa*, (**f**) *Eunicea tourneforti* and (**g**) *Plexaurella nutans*. Branch cross sections (in grey) drawn according to Cairns^42^. Photos are courtesy of Eric Jordán-Dahlgren.

**Figure 2:**
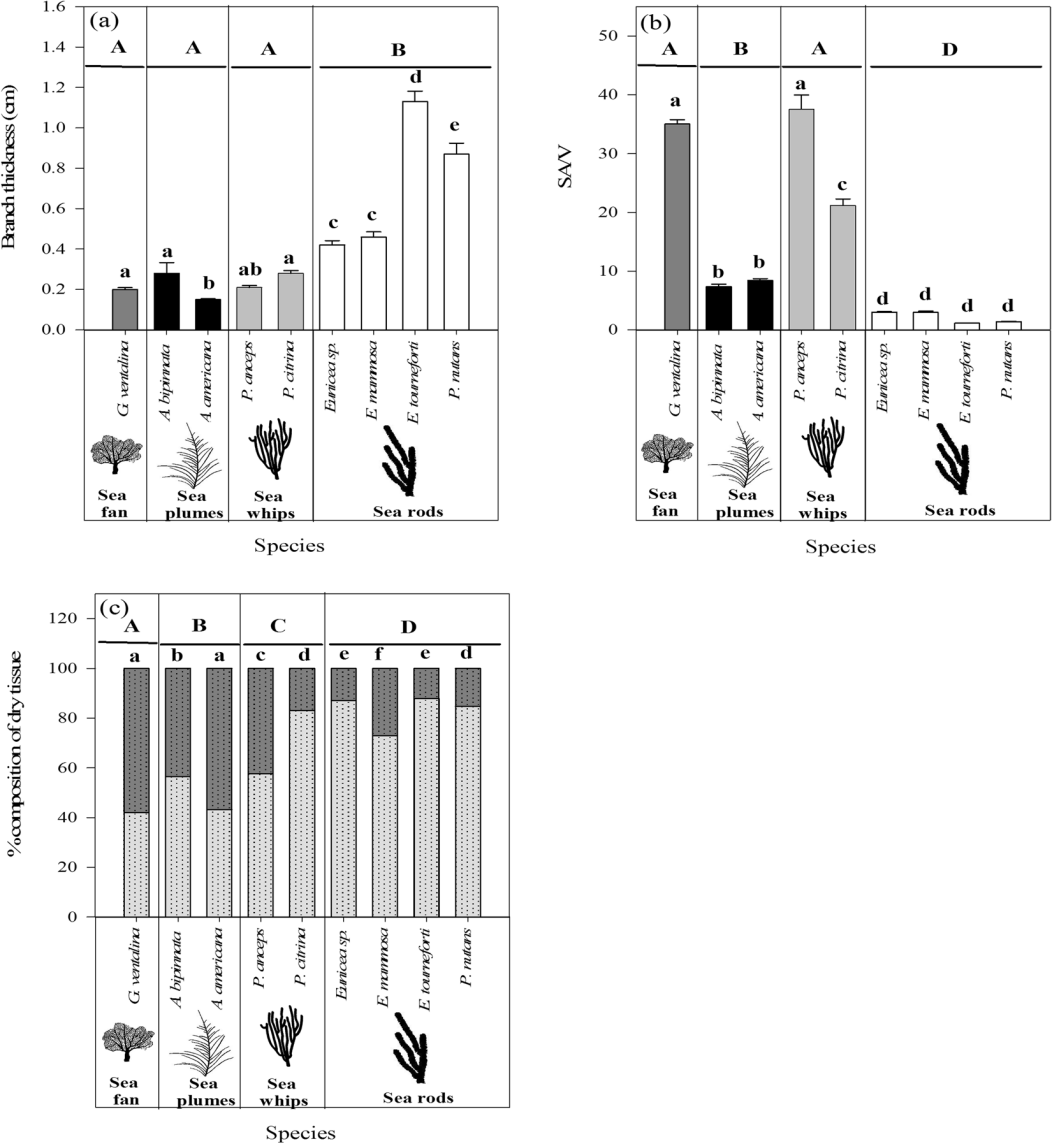
Comparison of the morphological characteristics of the studied Caribbean octocoral species: (**a**) branch thickness (n = 6 per species), (**b**) surface area to volume ratio (SA/V) (n = 10 per species) and (**c**) sclerite (light grey) and organic matter content (dark grey) (n = 6 per species). Results of one-way ANOVA to indicate significant differences (ANOVA, Newman-Keuls test, *p* < 0.05) between species and groups based on branch morphology are shown as lowercase and uppercase letters, respectively. Data represent means ± SE.

The species studied here represent a variety of colony morphologies and thus, differences in branch shape and thickness (Fig. 1). The branch thickness varied between species, but in general they could be divided into two groups, species with thin branches, which included the sea fan, plumes and whips, and those with significantly thicker branches, the sea rods (ANOVA, F_3,50_ = 25.9, *p* < 0.0001) (Fig. 2a). The species also differed in branch shapes, with some species, like the sea fan and whips, presenting more flattened branches, resulting in significantly higher SA/V, while the round branches of the sea rods exhibit the lowest SA/V (ANOVA, F_3,96_ = 255.3, *p* < 0.0001) (Fig. 2b). In addition, the studied species differed in micromorphological traits, such as the dimensions of their polyps (see Supplementary Table S1). This trait was related to the species’ macro-morphological features (SA/V, branch thickness). With decreasing polyp size, the colony SA/V of the species increased exponentially (Supplementary Fig. S1a), however, this correlation was not significant (R^2^ = 0.34, *p* = 0.075). In contrast, the branch thickness of the species showed a significant linear relationship with polyp size (Supplementary Fig. S1b; R^2^ = 0.94, *p* < 0.0001).

The species studied also differed significantly in organic and inorganic matter contents, with consistent patterns among species with similar colony morphologies. The lowest organic matter contents were found in sea rods, followed by the sea whips, while sea plumes and the sea fan exhibited the highest values (ANOVA, F_3,48_ = 76.9, *p* < 0.0001; Fig. 2c). The differences in inorganic matter content, which is mainly composed of sclerites, might be related to the significantly longer and wider sclerites found in sea rods, while the other species had smaller sclerites (Supplementary Table S2). On the other hand, the presence and amount of colored sclerites was species-specific and did not show a relationship with branch morphology (Supplementary Table S2). For example, while no colored sclerites were found in *E. tourneforti* and *P. nutans*, they represented a high proportion of the total sclerites in *E. mammosa* and *G. ventalina* (Supplementary Table S2).

Additional principal component analyses, including the aforementioned macro- and micro-morphological traits, supported the separation of the studied species into four groups, based on their common names, sea fan, plumes, whips and rods (Supplementary Fig. S1).

### Comparison of species’ photo-physiological performance

**Figure 3:**
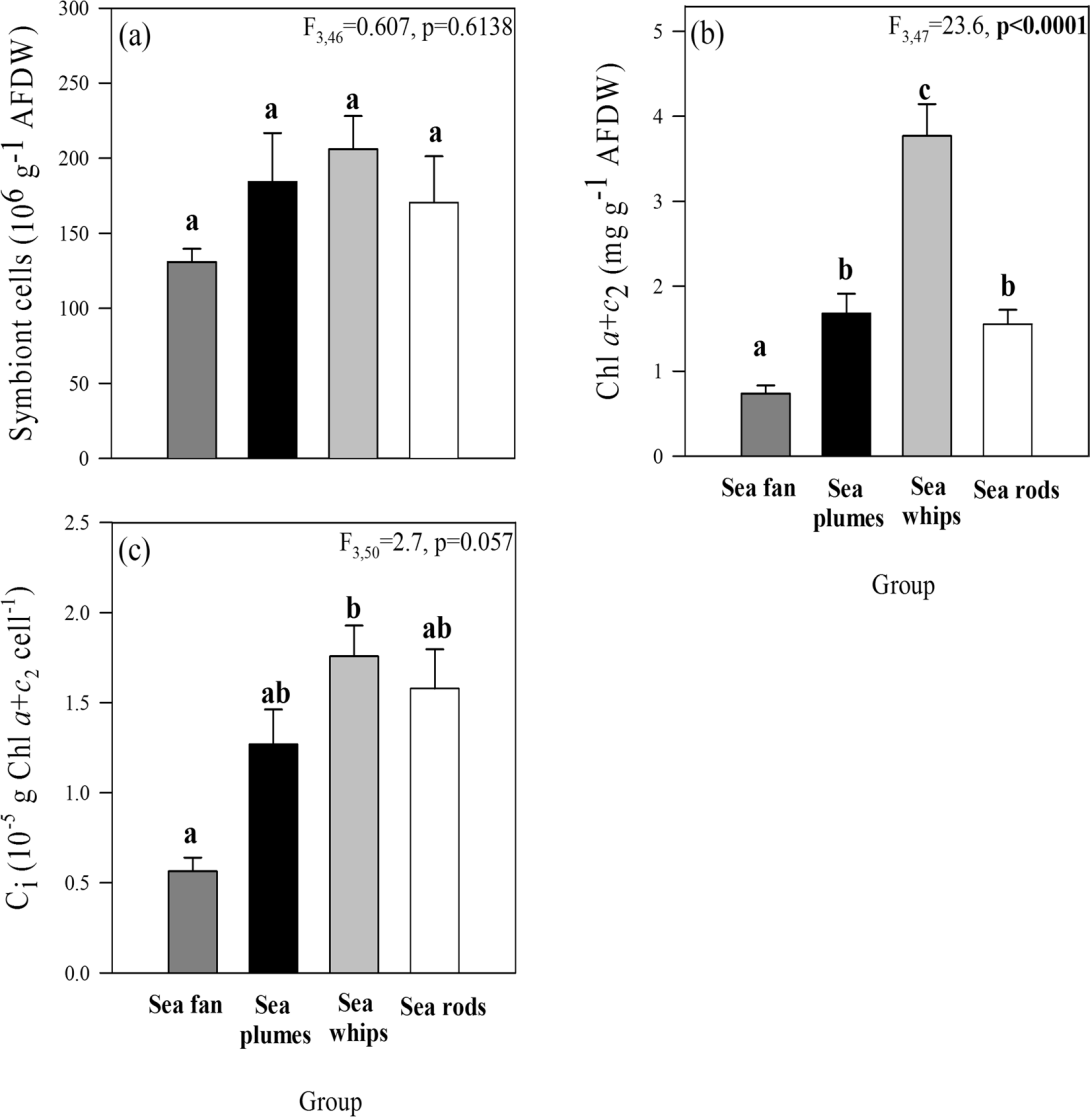
Differences in symbiont cell numbers and chlorophyll content, normalized by AFDW (**a**,**b**), and chlorophyll content per symbiont cell (**c**) of octocoral species grouped based on similar morphological traits. Results of one-way ANOVA are shown and significant differences between groups (ANOVA, Newman-Keuls test, *p* < 0.05) are indicated by superscript letters. Data represent means ± SE (n = 6).

No significant differences in symbiont cell density were found between the different branch morphologies (Fig. 3a), even though there were some differences between species (Supplementary Table S3). On the other hand, differences in branch morphology were accompanied by significant differences in chlorophyll content per ash-free dry weight (AFDW) (ANOVA, F_3,47_ = 23.6, *p* < 0.0001), with the sea whips and the sea fan exhibiting the highest and lowest chlorophyll contents, respectively (Fig. 3b). These differences resulted in the highest chlorophyll content per symbiont cell (C_i_) in the sea whips, while the sea fan *G. ventalina* showed the lowest C_i_ (Fig. 3c; Supplementary Table S3).

A correlation between branch morphology and the photosynthetic performance of the species was also found. The relationship between photosynthesis and irradiance was exponential in all species, with a coefficient of determination R^2^ > 0.95, showing differences between species, but in most cases a similar pattern for species with comparable morphological traits (Supplementary Fig. S3). The parameters derived from the P-E curves showed significant differences between species and branch morphologies, when either normalized to AFDW or symbiont cell number (Table 1) or surface area and chlorophyll content (Supplementary Table S4).

**Table 1:** Photosynthetic parameters in different octocoral species.

Parameter	Sea fan	Sea plumes		Sea whips		Sea rods				One-way ANOVA	
	*G. ventalina*	*A. bipinnata*	*A. americana*	*P. anceps*	*P. citrina*	*Eunicea* sp.	*E. mammosa*	*E. tourneforti*	*P. nutans*	F	*p*
**per g AFDW** ^**−1**^											
P_max_	301 ± 15^a^	210 ± 21^b^	182 ± 4^c^	306 ± 10^a^	77 ± 3^d^	48 ± 6^de^	35 ± 2^e^	28 ± 5^e^	68 ± 6^d^	139.4	**<0.0001**
α	0.49 ± 0.03^a^	0.26 ± 0.04^b^	0.32 ± 0.01^b^	0.46 ± 0.05^a^	0.12 ± 0.01^c^	0.07 ± 0.01^c^	0.06 ± 0.003^c^	0.04 ± 0.005^c^	0.1 ± 0.01^c^	63.4	**<0.0001**
R_D_	64 ± 8^a^	39 ± 10^b^	38 ± 2^b^	61 ± 3^a^	11 ± 1^c^	9 ± 1^c^	5 ± 1^c^	7 ± 1^c^	11 ± 1^c^	34.9	**<0.0001**
R_L_	85 ± 7^a^	56 ± 7^b^	57 ± 2^b^	85 ± 4^a^	18 ± 1^d^	15 ± 2^d^	10 ± 1^d^	10 ± 2^d^	16 ± 1^d^	88.5	**<0.0001**
**per cell** ^**−1**^ *10 ^**−6**^											
P_max_	2.4 ± 0.2^ab^	2.1 ± 0.4^ac^	2.9 ± 0.4^b^	1.5 ± 0.1 ^cd^	1.5 ± 0.2^d^	0.87 ± 0.01^d^	1.0 ± 0.1^d^	1.1 ± 0.1^d^	1.4 ± 0.1 ^cd^	11.35	**<0.0001**
α (x10^−2^)	0.41 ± 0.04^a^	0.28 ± 0.003^b^	0.47 ± 0.06^a^	0.21 ± 0.02^bc^	0.23 ± 0.02^bc^	0.12 ± 0.01^c^	0.22 ± 0.03^bc^	0.16 ± 0.03^bc^	0.23 ± 0.03^b^	15.07	**<0.0001**
E_c_	131 ± 17^abc^	156 ± 16^ab^	120 ± 5^ac^	138 ± 11^abc^	98 ± 5^c^	116 ± 16^ac^	88 ± 17^c^	172 ± 16^b^	112 ± 16^ac^	4.7	**0.00036**
E_k_	588 ± 28^a^	789 ± 56^a^	577 ± 27^a^	716 ± 53^a^	663 ± 23^a^	601 ± 82^a^	613 ± 48^a^	669 ± 71^a^	678 ± 51^a^	2.2	**0.0466**
P_int_	2.44 ± 0.2^a^	1.34 ± 0.2^b^	1.56 ± 0.02^b^	2.58 ± 0.2^a^	0.63 ± 0.02^c^	0.38 ± 0.05 ^cd^	0.26 ± 0.03^d^	0.22 ± 0.03^d^	0.54 ± 0.07^fcd^	95.6	**<0.0001**
R_(RL:RD)_	1.79 ± 0.2^a^	0.98 ± 0.12^b^	1.14 ± 0.04^b^	1.81 ± 0.06^a^	0.35 ± 0.01^c^	0.30 ± 0.02^c^	0.17 ± 0.02^c^	0.21 ± 0.04^c^	0.31 ± 0.02^c^	118.7	**<0.0001**
P_int_/R_(24h)_	1.7 ± 0.2^ab^	1.6 ± 0.2^b^	1.7 ± 0.1^ab^	1.7 ± 0.1^ab^	2.3 ± 0.1^a^	1.9 ± 0.3^ab^	1.8 ± 0.2^ab^	1.3 ± 0.1^b^	1.8 ± 0.2^ab^	3.50	**0.0035**
P_int_/R_(RL:RD)_	1.4 ± 0.1^abc^	1.3 ± 0.1^ab^	1.4 ± 0.1^abc^	1.5 ± 0.1^bc^	1.8 ± 0.02^d^	1.4 ± 0.1^abc^	1.2 ± 0.1^ab^	1.1 ± 0.1^a^	1.6 ± 0.2 ^cd^	6.39	**<0.0001**
H_sat_	6.25	3.5	6.25	4.25	5.0	5.0	6.25	5.5	5		

Data represent mean ± SE and significant differences between species (ANOVA, Newman-Keuls test, *p* < 0.05) are indicated by different superscript letters.

Maximum gross photosynthesis (P_max_), dark respiration (R_D_) and post-illuminatory respiration (R_L_) in μmol O_2_ h^−1^, α-photosynthetic efficiency in μmol O_2_ h^−1^ [μmol quanta m^−2^ s^−1^]^−1^, E_c_ and E_k_ - compensatory and saturating light intensity, respectively, in μmol quanta m^−2^ s^−1^, P_int_ (day^−1^) – daily integrated photosynthesis (see Fig. S4) (in mmol O_2_ g^−1^ AFDW), R_(24h)_ – daily integrated respiration, based on R_D_ (in mmol O_2_ g^−1^ AFDW), R_(RL:RD)_ – daily integrated respiration considering 12 h:12 h cycle for R_L_:R_D_ (in mmol O_2_ g^−1^ AFDW), P^/^R_(24h)_ - ratio of daily integrated photosynthesis (see Fig. S4) to 24 h R_D_, P/R_(RL:RD)_ – ratio of daily integrated photosynthesis to respiration (12 h:12 h cycle for R_L_:R_D_), H_sat_ – daily hours above average E_k_, considering light data from the field averaged over the sample collection month (September; see example in Fig. S4).

**Figure 4:**
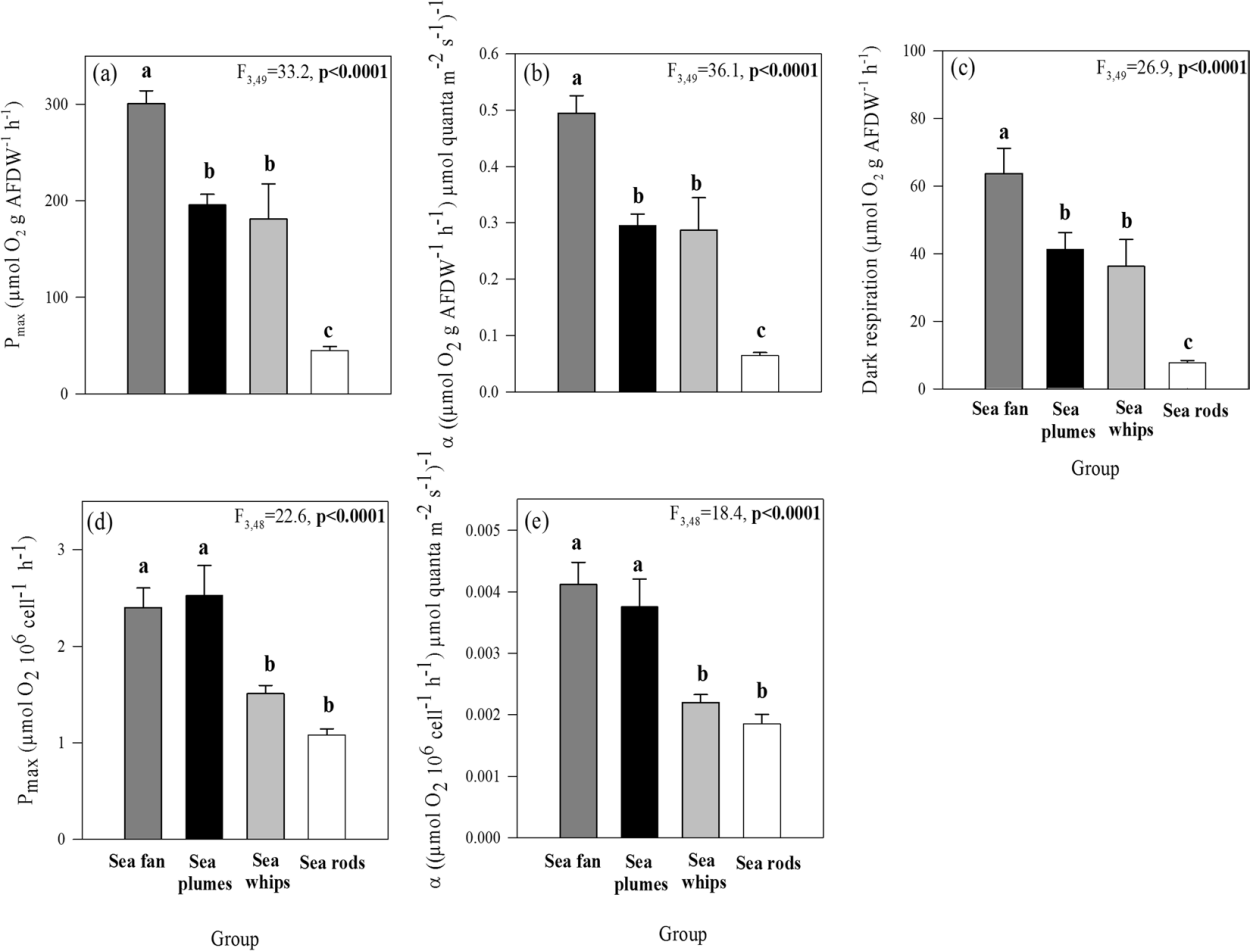
Differences in photosynthetic parameters, normalized by AFDW (**a**–**c**) and symbiont cell number (**d**,**e**) of octocoral species grouped based on similar morphological traits. Results of one-way ANOVA are shown and significant differences between groups (ANOVA, Newman-Keuls test, *p* < 0.05) are indicated by superscript letters. Data represent means ± SE (n = 6).

When comparing the photosynthetic performance of the species, we found slight differences, depending on the normalization (AFDW or symbiont cell density) in the cases of the sea plumes and rods. When normalized to AFDW, the sea fan *G. ventalina* had the highest photosynthetic capacity (P_max_) and efficiency (α), as well as the highest respiration among the studied species, while the sea rods exhibited the lowest values (Fig. 4). The sea plumes, however, showed intermediate values, statistically different from sea fan when normalized by AFDW, while they were not statistically different from the sea fan when normalized by symbiont cell numbers (Fig. 4). In the case of the sea rods, they expressed significantly lower metabolic rates when normalized to AFDW; however, per symbiont cell they were no different from the sea whips.

**Figure 5:**
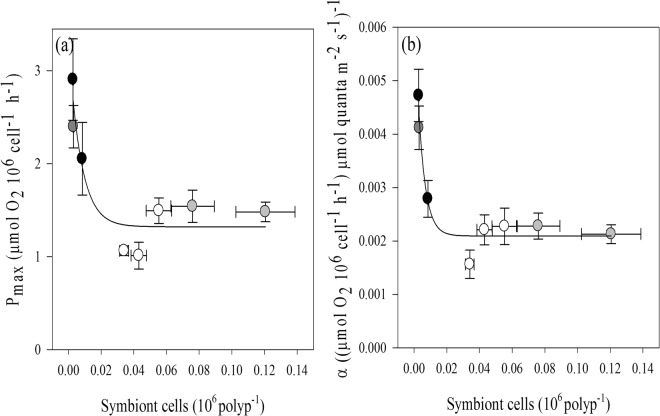
Relationships between the number of symbiont cells per polyp and the photosynthetic performance of the different octocoral species, normalized by symbiont density (sea fan- dark grey circle, sea plumes- black circles, sea whips- grey circles, sea rods- white circles). Exponential relationship between symbiont density and (**a**) maximum photosynthetic rate (R^2^ = 0.82, y = 1.32 + 1.99^(−133.2x)^, *p* = 0.0061) and (**b**) photosynthetic efficiency (R^2^ = 0.92, y = 0.0021 + 0.0046^(−230.5x)^, *p* = 0.0008). Data represent means ± SE (n = 6).

When plotting the symbiont cell density per polyp against the photosynthetic performance per symbiont cell, maximum photosynthesis and efficiency decreased exponentially with increased symbiont cell density (P_max_: R^2^ = 0.82, *p* = 0.0062; α: R^2^ = 0.92, *p* = 0.0008), with sea whips and rods having the highest cell density per polyp, also exhibiting the lowest P_max_ and α values (Fig. 5).

### Symbiont distribution

**Figure 6:**
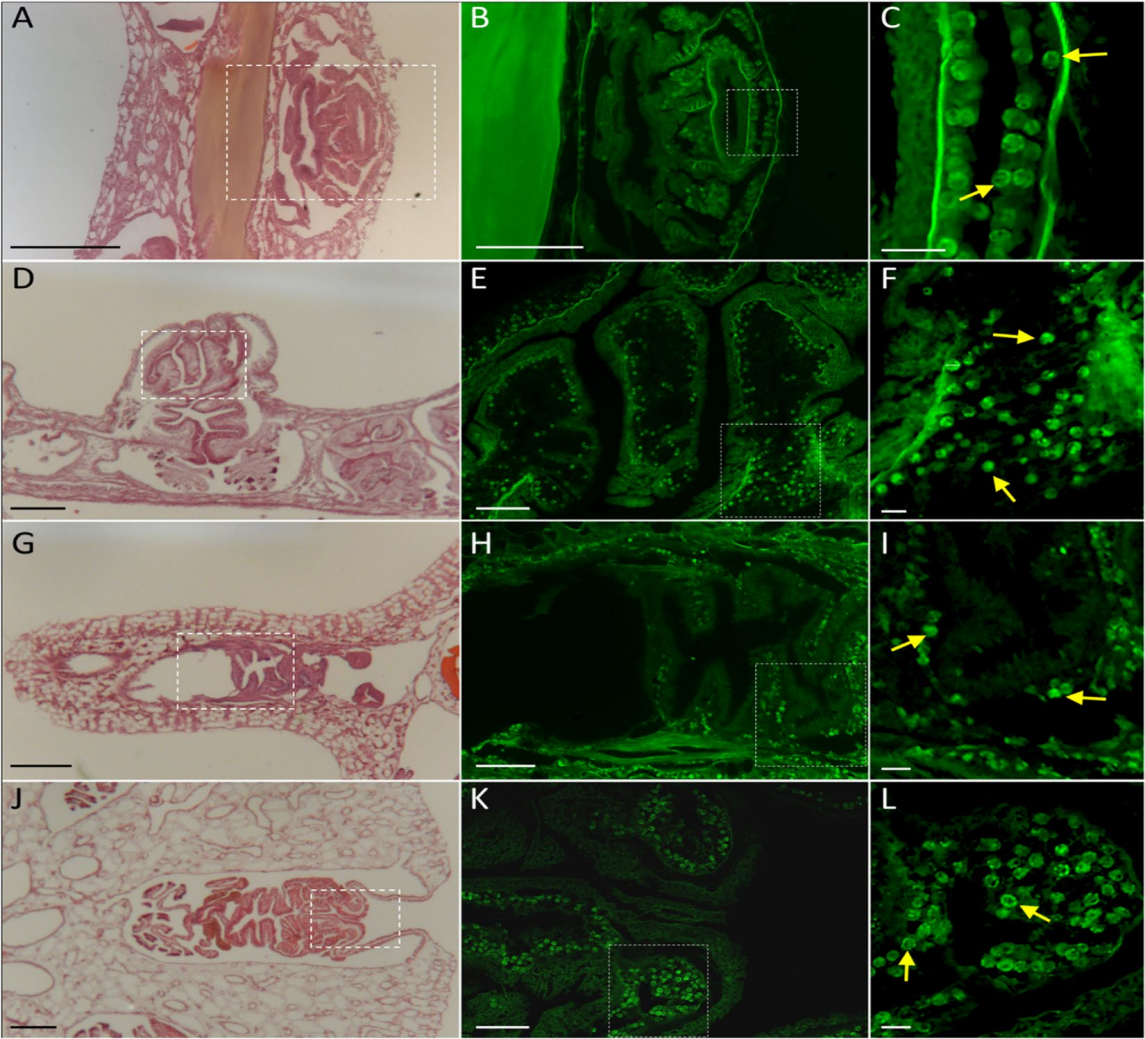
Symbiont distribution in gorgonian corals. Histological sections from (**A**–**C**) *Gorgonia ventalina*, (**D**–**F**) *Antillogorgia americana*, (**G**–**I**) *Pterogorgia anceps* and (**J**–**K**) *Plexaurella nutans*. (**A**, **D**, **G** and **J**) are sections stained with hematoxylin-eosin dyes and photographed at 10X amplification. (**B**,**E**,**H** and **K**) are the same sections but observed using an eGFP fluorescence filter (Ex488 – Em509 nm), the fluorescence labeling corresponds to Eosin. Dotted boxes in **A**,**D**,**G** and **J** represent the amplified regions shown in **B**,**E**,**H** and **K**, respectively. For even more zooming, dotted boxes in **B**,**E**,**H**, and **K** represent the amplified regions observed in **C**,**F**,**I** and **L**, respectively. Yellow arrows label symbionts. Scale bars are 100 µm (**A**,**D**,**G**,**J**), 25 µm (**B,E,H,K**) or 5 µm (**C**,**F**,**I**,**L**).

For *Gorgonia ventalina, Pseudopterogorgia americana, Pterogorgia anceps* and Plexaurella nutants, Symbiodinium cells were observed in octocoral branches both in the polyp and the coenenchyme (tissue that connects polyps), but a qualitative microscopic analysis showed these were much more abundant in polyps (Fig. 6, Supplementary Fig. S8).

### Symbiont photosynthesis versus host morphology

**Figure 7:**
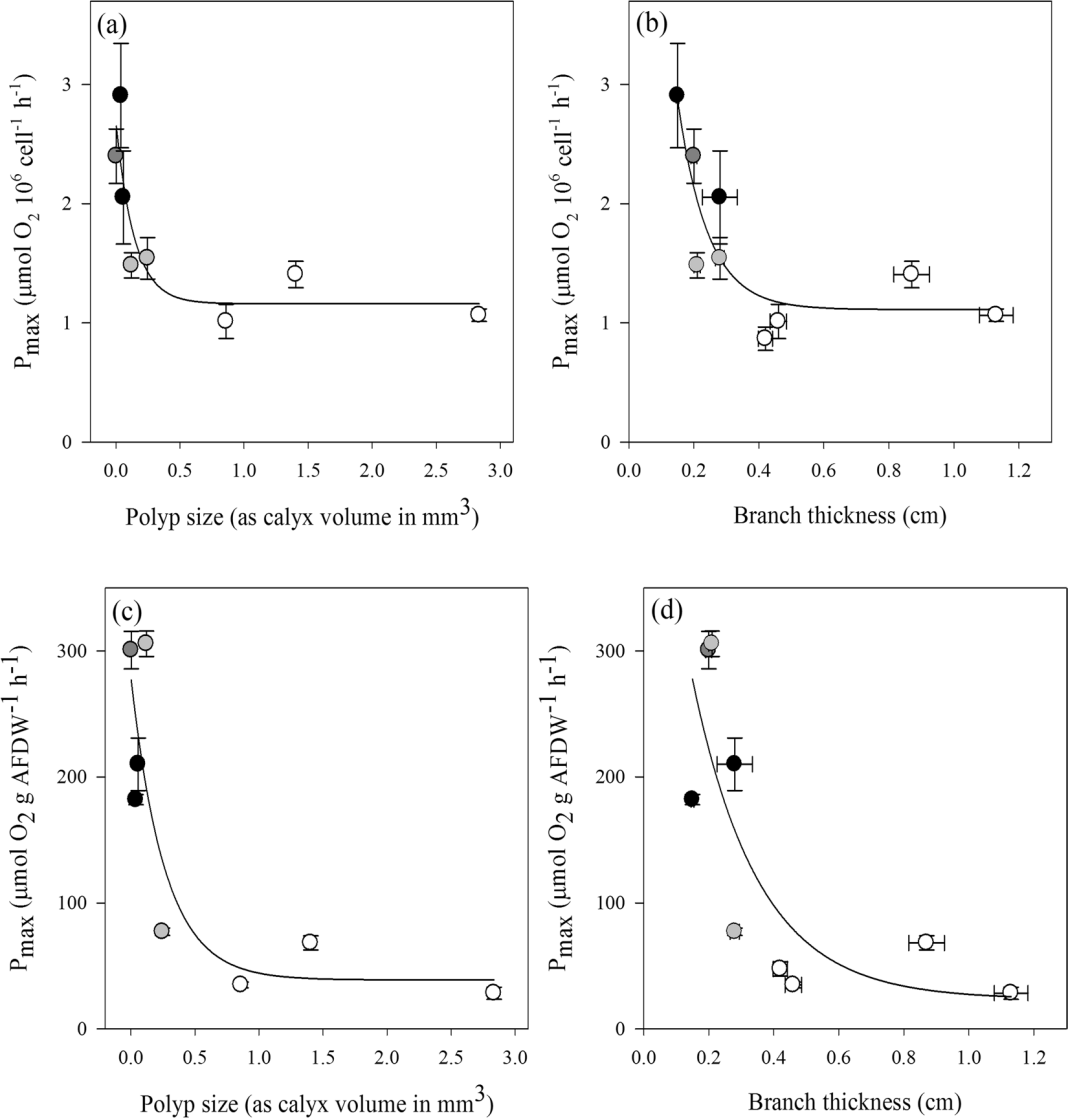
Effect of polyp size (**a**,**c**) and branch thickness (**b**,**d**) on the photosynthetic performance of octocoral symbionts, normalized to symbiont cell number (**a**,**b**) and AFDW (**c**,**d**) (sea fan- dark grey circle, sea plumes- black circles, sea whips- grey circles, sea rods- white circles). Data represent means ± SE (n = 6).

Maximum photosynthesis per symbiont cell decreased exponentially with polyp size (R^2^ = 0.71, *p* = 0.0197) and branch thickness (R^2^ = 0.69, *p* = 0.0120) (Fig. 7a,b). Similar results were also found when normalizing the maximum photosynthetic rate by AFDW, even though here the relationships were less significant (R^2^ = 0.65, *p* = 0.0304 against polyp size; R^2^ = 0.43, *p* = 0.0507 against branch thickness) (Fig. 7c,d).

**Figure 8:**
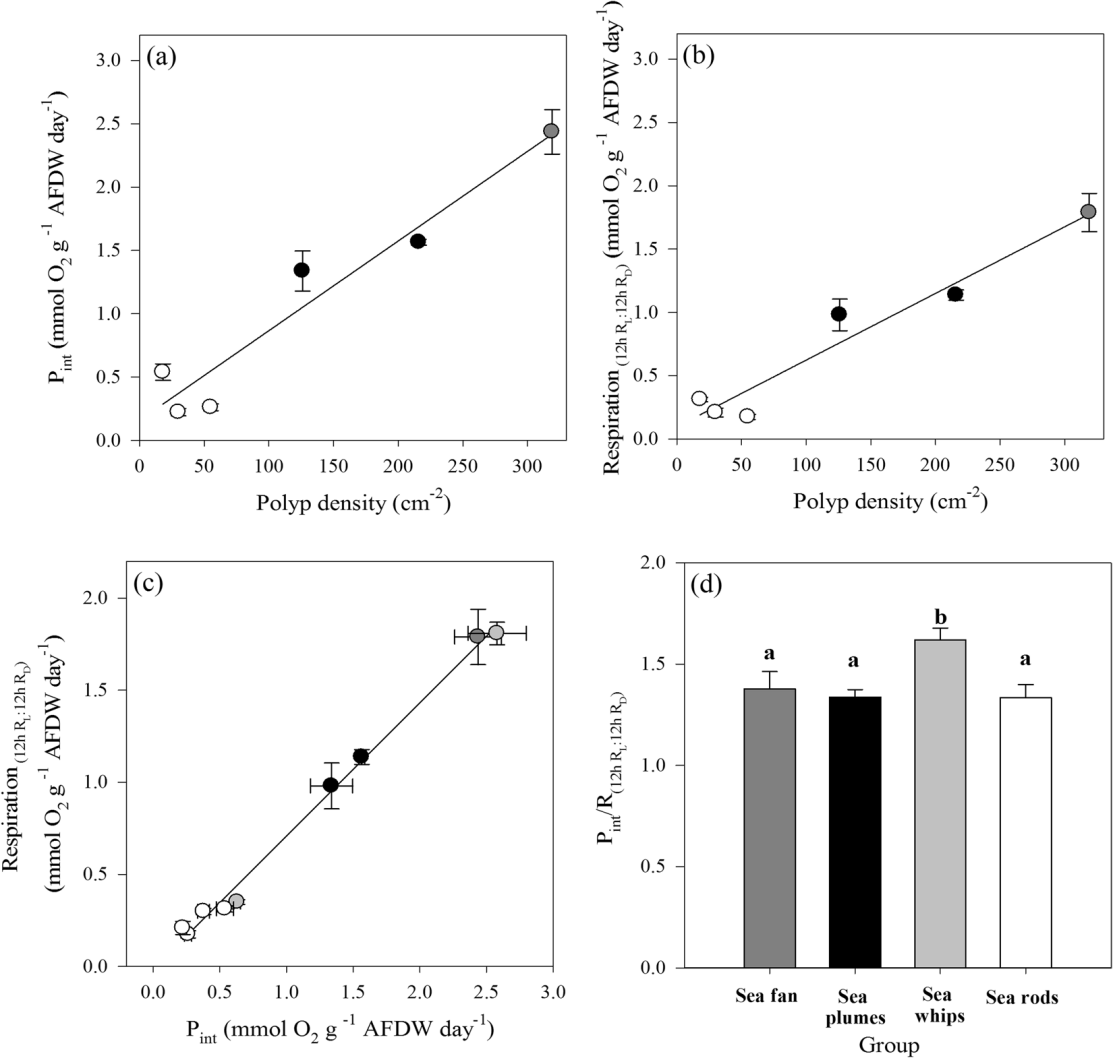
Linear relationships between polyp density and (**a**) daily integrated gross photosynthesis and (**b**) respiration (sea whips are not included here, due to their polyp arrangement in lateral series only along the elevated branch ridges). (**c**) Linear relationship between daily photosynthesis and respiration and resulting P/R ratios (**d**) for octocorals with different morphological traits (sea fan- dark grey, sea plumes- black, sea whips- grey, sea rods- white). For calculation of the daily integrated respiration and P/R ratio, light respiration (R_L_) to R_D_ over a 12 h: 12 h cycle was considered. Results of one-way ANOVA and posthoc test are shown and significant differences between groups (ANOVA, Newman-Keuls test, *p* < 0.05) are indicated by superscript letters. Data represent means ± SE.

The daily integrated photosynthesis and respiration, when normalized by AFDW showed a significant linear relationship with the polyp density per surface area (Fig. 8a,b), both increasing with polyp density (P_int_: R^2^ = 0.91, *p* = 0.0018; Respiration: R^2^ = 0.93, *p* = 0.0012). In addition, daily photosynthetic and respiratory rate of the species were directly related, showing that species that exhibited the highest photosynthetic rate, such as the sea fan *G. ventalina*, also had the highest respiratory rate (Fig. 8c¸ R^2^ = 0.99, *p* < 0.0001). The resulting photosynthesis to respiration ratio (P/R), with values >1 (from 1.1 to 1.8) in all species, showed significant differences between groups (ANOVA, F_3,45_ = 4.2, *p* = 0.01134). The sea whips reached significantly higher ratios compared to the other groups (1.5–1.8; Table 1), while there were no significant differences between sea fan, plumes and rods (Fig. 8d).

#### Stable isotopes

The δ^13^C values of the nine studied species varied between −13.8‰ in *P. anceps* and −17.7‰ in *A. americana* (Supplementary Fig. S4). The values of the δ^15^N stable isotope oscillated between a minimum of 4.2‰ in *P. nutans* and a maximum of 7.5‰ in *E. mammosa* (Supplementary Fig. S4).

## Discussion

The present study shows that octocoral symbiont performance is either directly or indirectly correlated to certain host morphological traits and that higher symbiont performance does not necessarily translate to a higher autotrophic input to the hosts’ energy budget.

On one hand, symbionts are concentrated in the polyps (see Fig. 6^16,17^); and thus, our findings of direct correlations between polyp size and density and symbiont photosynthetic performance are not surprising. On the other hand, polyp size of the studied species is directly related to branch morphology (Supplementary Fig. S1), which is why we also found an indirect correlation between symbiont photosynthetic performance and this morphological trait.

The direct correlation between symbiont photosynthesis and polyp size was most likely related to the fact that the latter defines polyp SA/V, and hence, light absorption efficiency and gas exchange. For example, species like the sea fan *G. ventalina* and the sea plumes *Antillogorgia* spp. possess very small polyps compared with sea rods, whose polyps are more than an order of magnitude larger (Supplementary Table S1). As a consequence, the polyp density and SA/V of each polyp, relative to the overall colony size, is much higher in sea fans and plumes. This fact, together with lower symbiont cell densities per polyp, increases the amount of light reaching the symbionts in the polyp tissues, and hence, their photosynthetic performance (Fig. 5). The converse appears to be true for most sea rods, where larger and fewer polyps, higher symbiont density per polyp, and the thick, rod-shaped branch morphology seems to limit productivity, both in terms of per unit of symbiont cell and per AFDW. Larger polyps might result in more efficient feeding structures; however, several studies on non-symbiotic species also reported that gorgonians bearing the smaller polyps did not show smaller prey size or lower capture rates^18,19^.

The sea whips represent an exceptional case, as their polyps, which are of intermediate size, have a very low density per surface area due to their arrangement in lateral series, only along the elevated branch ridges^20^. This, in addition with a very high symbiont cell density per polyp (1.5–2.4 and 10–29 times higher compared to sea rods and sea fan and plumes, respectively), seems to limit the photosynthetic performance of these species, most likely due to self-shading (“package effect”; *sensu*^21^) between symbiont cells.

In the species studied here, polyp size showed a strong association with branch thickness (Supplementary Fig. S1b), which seems to be a general feature in octocorals^22^. Hence, symbiont photosynthesis was also found to be correlated with branch morphology, resulting in higher photosynthetic capacity per symbiont cell in sea fans and plumes, compared to sea whips and rods (see Fig. 4d). This might be in part due to the alternating and sometimes very flexible branches^10^ of sea plumes that could aid in reducing self-shading. Sea fans, such as *G. ventalina*, have a net-like planar morphology with high surface area, which is positioned perpendicular to water flow^23^. This orientation, together with the flexibility of the colony due to relatively low sclerite content, facilitates the oscillation of the colony from full illumination to shade in synchrony with the wave period, thus maximizing symbiont performance, a feature that they share with the sea plumes. The movement of the bio-structures (like the leaves of marine phanerogams) has been demonstrated to be a key point to understand the optimization of light harvesting within a canopy^24^, and is a parameter that may also be important in octocorals gathered in dense patch structures.

Our findings of higher photosynthetic performance when polyps are smaller partially agrees with the hypothesis put forth by Porter^8^. He reported an inverse relationship between SA/V of the colony and polyp size in Caribbean scleractinian corals and interpreted it in terms of the corals’ dependence on light-capturing ability versus plankton-capturing ability, respectively, assuming that small polyp forms are ineffective at prey capture and better suited for light capture. In the octocorals studied here, a similar relationship between SA/V and polyp size was found as reported by Porter^8^ (see Supplementary Fig. S1a) and species with smaller polyps had a higher photosynthetic capacity. Interestingly, in contrast to Porter’s conclusion, it did not translate into a more or less autotrophic nature of the species. In all species, the P/R values, derived from the daily integrated photosynthetic and respiratory rates of the species, were >1, indicative of at least partial reliance on autotrophic input for their energy demands. Despite its high photosynthetic rates, the sea fan *G. ventalina* did not exhibit higher daily net production, as it also had the highest respiratory rates. This seems to be a common feature of sea fans, also reported previously by Lewis and Post^25^, whom reported two times higher respiration rates in sea fans compared to sea rod species (Supplementary Table S5), most likely related to their high polyp density. Thus, the higher respiratory rates in species with smaller polyps and higher polyp density (sea fan, plumes) translated to similar P/R ratios as in species with larger polyps, such as the sea rods (see Fig. 8d). On the other hand, in sea whips, with polyps of intermediate sizes, the contribution of symbiont photosynthates to the hosts’ energy budget seemed to be potentially more important than in the other species (see Fig. 8d).

This inconsistency with Porters’ model might be related to: (1) not considering that smaller polyp size usually relates to higher polyp density, which might result in higher respiratory rates and (2) his assumption of a relatively uniform distribution of polyps over the surface area, which does not apply for the studied sea whips. In addition, previous studies showed that having smaller polyps per unit biomass of coral increases surface area for feeding or light capture^26,27^. The results of the δ^15^N analyses (Supplementary Fig. S4) indicate a species-specific, but nontrivial input of energy from a heterotrophic source^28^, which shows that there is not a strong relationship between polyp size and potential prey capture (see below).

**Figure 9:**
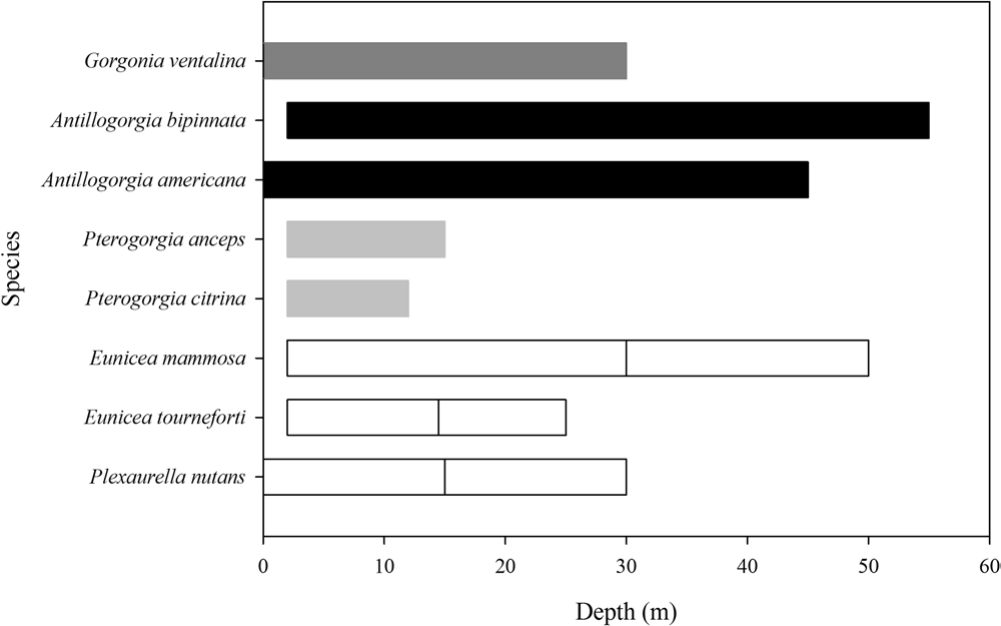
Reported depth ranges of studied Caribbean octocoral species (sea fan- dark grey, sea plumes- black, sea whips- light grey, sea rods- white), according to Kinzie^43^ and Goldberg^44^. Reported upper depth limits (>2 m) have been modified, as all species in this study were found at 2 m depth in the Puerto Morelos reef lagoon, Mexican Caribbean.

The higher potential autotrophic contribution in sea whips seems reasonable, as they have reduced polyp numbers, a trait that reduces self-shading between polyps but also diminishes the effectiveness of zooplankton feeding. This result also agrees with the reported shallower depth limit for the sea whips (≤15 m), ensuring high light availability, while sea fan, plumes and rods that potentially rely less on autotrophic input are able to extend their range deeper (Fig. 9).

In general, the comparison of P/R ratios between studies is difficult since photosynthesis and respiration determinations, as well as the way the ratios are calculated, differ greatly. In Red Sea octocorals, P/R values ranging from 1.0 to 1.4 have been found^29^, while they were slightly higher (1.07–1.61) in octocorals from the Great Barrier Reef^13^. The study of Baker *et al*.^15^ reported P/R of 0.71–1.87 in Caribbean octocorals, with the lowest values found in sea rod species, while sea fans achieved the highest P/R. In the shallow-water Caribbean octocorals studied here, the P/R values were slightly higher, ranging from 1.1 to 1.8 (see Table 1). The discrepancy with the results of Baker *et al*.^15^ might be due to differences in the way the photosynthetic and respiratory rates were measured and calculated. The P/R values reported in the former study derived from significantly lower metabolic rates, specifically lower photosynthetic rates, when compared to our study (Supplementary Table S5). This might have been the result of using freshly fragmented colonies and very long incubation times (7.5–8 h) in a small water volume (0.5 L), without stirring. This could have caused an underestimation of photosynthetic rates due to increased respiration of stressed samples, oxygen oversaturation and carbon limitation. Also, our calculations for P/R were done differently. We used an approach to obtain accurate and comparable daily integrated P/R ratios without the need of performing *in situ* measurements. For this, we used (a) light data from the field and the maximum photosynthetic capacity (P_max_) and the photosynthetic efficiency (α) of the species (derived from photosynthesis-irradiance curves) to calculate their daily integrated photosynthesis, and (b) light respiration values to account for the higher respiration during the day. We believe that the data obtained from our approach are comparable to those derived from *in situ* measurements over a 24-h cycle.

Our results of P/R >1 indicate that the autotrophic contribution to the octocorals’ energy budget is potentially important. However, the stable isotope values of the studied species (Supplementary Fig. S4) indicate that in neither of them the heterotrophic input is negligible^6,28^. Baker *et al*.^28^ showed that those areas in which eutrophic disturbances were detected, the δ^15^N values were as high as 7.7‰ in *G. ventalina* (very similar to those found in the present study), suggesting sewage-derived N inputs. Thus, it is possible that the conditions in the Puerto Morelos reef lagoon (no longer oligotrophic due to the high tourist pressure)^30^ allow a substantial N input from different heterotrophic sources^28^, thus coupling the autotrophic contribution with significant heterotrophic input. The heterotrophic input in Caribbean gorgonians is still a pending issue, being a potential factor that would help to understand the trophic flexibility of some species.

A final consideration to bear in mind is that despite the strong correlation between symbiont performance and species’ morphological traits and the related differences in the potential autotrophic contribution to hosts’ energy demands, other factors might have had an influence on our results.

One potentially important factor is related to the symbiont phylotype hosted by the species. Differences in the associated phylotype can affect symbiont performance and P/R, as it is well known in scleractinian corals that photosynthetic performance and the proportion of photosynthetate translocated to the host can differ greatly between phylotypes^31,32^. In the studied species, the degree of symbiont specificity varies from sea fan (B1) > sea plumes and whips (B1, B1i, B1m) > sea rods (B1, B1a, B9, B19, B41)^33,34^, which may have some influence on the species-specific differences in symbiont performance and P/R found here. In this context, Baker *et al*.^15^ pointed out that there might be a correlation between phylogeny and the species’ reliance on autotrophic input, as they found a strong negative correlation between polyp size and carbon translocation from the symbiont to the host. Thus, they suggested that selection might have favored more obligate associations with highly productive *Symbiodinium* in species that in turn have reduced feeding structures but increased SA/V for light capture (smaller polyps).

## Conclusions

Physiological studies in octocoral are scarce. The works investigating their physiological response to climate change^4^ are also very few and still pose more questions than answers. This study demonstrates that there exists a pattern between hosts’ morphological traits and symbiont performance and shows that there might be potential differences in the importance of the symbiosis for species’ energy budget. It also demonstrates that both heterotrophy and autotrophy have a clear role in the energy inputs, although there is still work to be done on their respective contributions in the octocorals’ mixotrophic strategy.

The general assumption of high resistance of this group to ocean warming, compared to scleractinian corals, has been attributed to their ability to lower the dependency on autotrophic input and increase the contribution of the organisms’ energetic demands through heterotrophy^13,35^. This might be the case in some of the studied species. But the potential higher dependency on autotrophy found here in sea whips, together with their low polyp density (suggesting inefficiency for heterotrophic feeding) leads us to hypothesize that these species might be more susceptible to increases in temperature in oligotrophic (non-perturbed) zones. The hypothesis is supported by a study from Puerto Rico, reporting the proportion of bleached colonies of different species in octocoral communities^36^. It showed that *Pterogorgia* sp. was one of the most affected species (84.5% bleached colonies), while species, such as *Gorgonia* sp., *Eunicea* sp. and *Antillogorgia* sp., were unaffected. The high resilience of Caribbean sea rod species to increased temperature has also been reported recently by Goulet *et al*.^34^, suggesting that these species are possibly more resistant to fast changing environmental factors. Thus, further work on the importance and efficiency of symbiont-host translocation in octocorals, as well as their flexibility in the relative contribution of autotrophy versus heterotrophy to octocoral nutrition, will be essential to shed light on the potential costs of the symbiosis, and hence, species’ resilience in the face of stressful conditions and associated possible future seascape transformations.

## Material and Methods

### Study site and sample collection

During September 2014, nine species of octocorals, representing a variety of colony morphologies, including a sea fan (*Gorgonia ventalina*), plumes (*Antillogorgia bipinnata, A. americana*), whips (*Pterogorgia anceps, P. citrina*) and sea rods (*Eunicea* sp., *Eunicea mammosa*, *E. tourneforti*, *Plexaurella nutans*) (Fig. 1) were sampled at ∼2 m depth in the Puerto Morelos reef lagoon, Mexican Caribbean (see^37^ for a detailed description of the study site). These species are considered abundant, key components of Caribbean coral reefs^3,10^. For each species, branches of six different colonies (5–8 cm height) were collected the day before the physiological measurements. The time elapsed between sampling and transport to the mesocosm facility at Universidad Nacional Autónoma de México (UNAM) did not exceed 15 minutes. The branches were fixed with non-toxic rubber in seawater flow-through tanks to allow recovery from handling. Preliminary tests showed that one day of recovery from sampling ensured adequate physiological conditions of the gorgonians, as the obtained photosynthetic rates were not different from samples kept to recover for two and three days before photophysiological measurements.

### Photosynthesis measurements

To determine oxygen fluxes of the octocorals, branches were incubated in a sealed temperature-controlled acrylic dual-chamber at 29 °C (temperature in the reef lagoon during collection period). The incubations were performed by exposing the samples to increasing light intensities and recording the changes in oxygen concentration through an optode system (FireStingO_2_, Pyroscience, Aachen, Germany), which in turn was connected to a computer running the Pyro Oxygen Logger (FireSting Pyroscience, Aachen, Germany) (see Supplementary Material for further details). Gross photosynthetic rates were calculated by adding respiration (average between dark and light respiration) to net photosynthesis. The highest photosynthetic rate was considered as P_max_ and the photosynthetic efficiency (α) was estimated from the initial slope of the light-response curve by linear least-square regression analysis. The irradiance at the onset of saturated photosynthesis (E_k_) was obtained from the ratio P_max_/α. For comparison with other studies, the photosynthetic parameters were normalized by ash-free dry weight (AFDW), symbiont cell number, surface area and chlorophyll *a* concentration of the samples. Using the photosynthetic efficiency (α) and daily variation of the irradiance at collection depth, daily integrated gross photosynthesis and respiration (Supplementary Fig. S5), as well as the photosynthesis to respiration ratios (P/R) were calculated (see Supplementary Materials for further details).

The branches used in the incubations were photographed next to a ruler (a small transversal cut was also made to obtain the diameter of the branches) to calculate the total surface (for further details on surface area and volume calculation see Supplementary Materials). Immediately afterwards, the samples were frozen at −80 °C for subsequently determination of symbiont density and chlorophyll concentrations and ash-free dry weight per area (see Supplementary Figure S6).

### Determination of chlorophyll concentrations and symbiont cell density

*Symbiodinium* cell densities and chlorophyll (Chl *a* and c_2_) concentrations were determined by adding filtered seawater to the tissue samples to a final volume of 5 mL, homogenizing them with an Ultra-homogenizer, after which the samples were centrifuged at 1500 x g for 15 min. The supernatant was discarded and the pellet resuspended in 5 mL filtered seawater, while subsamples were taken for subsequent cell density and chlorophyll determinations.

To determine chlorophyll concentrations, the pigment extraction was performed as outlined by Iglesias-Prieto *et al*.^38^ and chlorophyll *a* (Chl *a*) and *c* (Chl *c*_2_) contents were calculated using the equations of Jeffrey and Humphrey^39^. Chlorophyll concentrations were standardized to ash-free dry weight, branch surface area (see Supplementary Material) and *Symbiodinium* cell density.

A subsample of 1 mL was taken from the resuspended tissue pellet, fixed with Lugol’s solution and subsequently, the symbiont cells were counted using an improved Neubauer haemocytometer and normalized to ash-free dry weight and surface area.

### Octocoral sclerites and organic matter content

Determinations of the sclerite size and shape, as well as the proportion of colored sclerites were performed on sub-samples (n = 3 per species) that were placed in 1.5 ml Eppendorf tubes to which 5% sodium hypochlorite solution (household bleach) were added to remove the organic matter. After removing the supernatant, distilled water was added to the samples, and after stirring, an aliquot was placed on a slide and photos were taken from the sclerites (see Supplementary Fig. S7), using a binocular microscope (AxioImager Zeiss Microscope). Based on the photos, the length of the sclerites (n = 10 of each photo), as well as their maximum and minimum width were determined. In addition, the proportion of colored (pink to dark-brown) sclerites relative to the total amount of sclerites was determined from the photos.

For ash-free dry weight determination, branch fragments of 1 cm length from the samples used for photosynthetic measurements were subsampled from each species and photographed to calculate their area. Afterwards, the proteinaceous axis of the fragments was removed and ash-free dry weight was determined as described in Rossi *et al*.^40^. Based upon the ratio of AFDW per area, a conversion factor was determined for each species, which allowed the normalization of the photosynthetic parameters to metabolically active tissue (see Supplementary Figure S6).

### Symbiont distribution in octocoral tissue

For histological analysis, octocoral branches were fixed in formalin (10%) and treated in a series of rinsing and decalcification steps (for further details see Supplementary Materials), after which the tissue sections were stained with Meyer’s hematoxylin and eosin procedures, and coverslipped with Organol/Limonene (Sigma O8015) mounting medium. Subsequently, photographs were obtained using an AxioImager Zeiss Microscope.

### Stable isotope analysis

Proportions of carbon (δ^13^C) and nitrogen (δ^15^N) stable isotopes can vary with nutrient source and trophic level of consumers. Thus, stable isotope analysis has been successfully used to elucidate food source partitioning, and food web dynamics^41^. Samples of octocoral coenenchyma (n = 5 for each species) were oven-dried at 60 °C and homogenized using mortar and pestle. To remove carbonate structures, the sample was acidified by adding 1 M HCl drop-by-drop^41^. Afterwards, the samples were re-dried at 60 °C for 24 h.

One to five milligrams per sample were weighed in tin capsules, combusted and the resultant gases were analyzed in a Thermo Electron Isotope Ratio Mass Spectrometer for stable isotope abundance. Internal laboratory standards were used after every five samples to calibrate the system. Isotopic values were expressed in δ notation as parts per thousand (‰) differences from international standards (Vienna Pee Dee Belemnite and atmospheric N_2_ for carbon and nitrogen respectively): δ*X* = [(*R*_sample_/*R*_standard_) − 1] * 10^3^, where *X* is equal to ^15^N or ^13^C and *R* is the corresponding ratio ^13^C/^12^C or ^15^N/^14^N. Based on replicates of laboratory standards, analytical precision was ±0.2 and ±0.1‰ for δ^15^N and δ^13^C, respectively.

### A posteriori tests

Since most of the symbionts reside in the polyps (this study^16,17^), we tested the correlation between symbiont photosynthesis and cell density per polyp. To that end, we used the data on intercalyx distance reported by Velásquez and Sánchez^11^ for the studied species to calculate the number of polyps per surface area (Supplementary Table S1), which allowed us to calculate the number of symbiont cells per polyp based on our data of symbiont cell density per surface area. To test our hypothesis that symbiont physiology correlates to the species’ morphological features, we also used macro-morphological data (branch thickness) and micro-morphological data (polyp size) to show the relationship between these morphological traits and symbiont photosynthesis.

As proxy for polyp size, the volume of the calyx (structure into which the polyp retracts) was used, calculated from data on calyx depth and diameter, reported by Velásquez and Sánchez^11^ for the studied species (Supplementary Table S1). Either calyx/polyp diameter or depth (distance from the base where polyp is anchored to surface edge of aperture) have been used in previous studies as a proxy for polyp size. However, there is no clear relationship between calyx diameter and depth, as similar diameters could be found in sea whips and rods, but when considering also the depth of the calyx, the calculated space that the polyp occupies, and therefore polyp size (here calculated as calyx volume), differed greatly between these two groups (Supplementary Table S1). Thus, we consider that including both features, as a proxy for the anatomical space the polyp is occupying, represents a more exact proxy for polyp size.

### Statistical analysis

Data were tested for normality using the Shapiro-Wilk test, and for equal variance using the Levene median test. Analyses of variance (one-way ANOVA) allowed for the determination of significant differences (*p* < 0.05) for each parameter, with octocoral species or groups based on morphological traits as independent factors. For the comparison of differences between species and groups, a Newman-Keuls (NK) Post-hoc test was used. The statistical analyses were conducted using Statistica 12.0. To determine species’ grouping based on morphological traits, principal component analysis (PCA) was performed using R.

## Electronic supplementary material

Supplementary Material
