# Supplementary material for: Linking host morphology and symbiont performance in octocorals
Source: Sci Rep. 2018 Aug 27;8:12823. doi: 10.1038/s41598-018-31262-3 (PMC6110782; doi:10.1038/s41598-018-31262-3)
Supplement: Supplementary file 1 — Supplementary Material [file 41598_2018_31262_MOESM1_ESM.doc]

**SUPPLEMENTARY MATERIAL OF**

**Linking host morphology and symbiont performance in octocorals**

Sergio Rossi^1,2,a,*,+^, Nadine Schubert^2,b,+^, Darren Brown^3^, Marcelo de Oliveira Soares^1,4^, Victoria Grosso^2^, Emma Rangel-Huerta^2^ and Ernesto Maldonado^2^

^1^ The Environmental Science and Technology Institute, Autonomous University of Barcelona, Campus UAB s/n, Barcelona, 08193, Spain

^2^ Unidad Académica de Sistemas Arrecifales Puerto Morelos, Instituto de Ciencias del Mar y Limnología, Universidad Nacional Autónoma de México, Puerto Morelos, 77580, Mexico

^3^ Department of Biology, The Pennsylvania State University, University Park, Pennsylvania, 16802, USA

^4^ Instituto de Ciências do Mar (Labomar), Universidade Federal do Ceará, Fortaleza, 60165-081, Brazil

^a^ Current address: DiSTeBA, University of Salento, Lecce, 73100, Italy

^b^ Current address: Programa de Pós-graduação em Oceanografia, Centro de Ciências Físicas e Matemáticas, Universidade Federal de Santa Catarina, Campus Trindade, Florianópolis, 88040-970, Brazil

^*^ Corresponding author: [sergio.rossi@unisalento.it](mailto:sergio.rossi@unisalento.it)

^+^ These authors contributed equally to this work.

**Figure S1**. Relationships between the polyp size of the studied species and their (a) SA/V (R^2^=0.34, y=24.1(-1.45x), p=0.075) and (b) branch thickness (R^2^=0.94, y=-0.51+2.69x, p<0.0001) (sea fan- dark grey, sea plumes- black, sea whips- grey, sea rods- white). Data of polyp size according to Velásquez and Sánchez (2015) (see Table S4).

**Figure S2**. PCA analysis based on macro- and micro-morphological traits of the species (sea fan- green, sea plumes- violet, sea whips- blue, sea rods- black).

**Figure S3**. Photosynthesis vs irradiance curves of the different octocoral species, grouped based on similar morphological traits: a & e- sea fan; b ; f- sea plumes; c & g- sea whips; and d & h- sea rods. The data were normalized by ash-free dry weight (a-d) and symbiont cell number (e-h). Data represent means ± SE (n=6).

**Figura S4.** Proportions (‰) of stable isotopes of carbon (δ^13^C) and nitrogen (δ^15^N) in the studied octocoral species (mean ± SD).

*Photosynthesis measurements*

The incubations were performed with 0.45 μm filtered seawater and NaHCO_3_ was added for a final concentration of 4 mM to prevent CO_2_ limitation during incubations. Each chamber was equipped with an optode fiber optic that was connected to the optical oxygen meter (FireStingO_2_, Pyroscience, Aachen, Germany), which, in turn, was connected to a computer running the Pyro Oxygen Logger (FireSting Pyroscience, Aachen, Germany). Optodes were calibrated against nitrogen-saturated and air-saturated seawater for the 0% and 100% oxygen, respectively. Before starting the incubations, the oxygen concentration in the seawater was lowered to approx. 50% through bubbling with nitrogen, to prevent inhibition of photosynthesis via photorespiration.

Following a 15-20 min acclimation period, respiration was measured for 15 min in darkness (R_D_). Afterwards, the branches were illuminated with 3 W LED light bulbs from two opposite directions and exposed to 12 progressively increasing irradiance levels (0 to 1400 μmol quanta m^-2^ s^-1^). Each light level was imposed for approximately 10-15 min, enough time to obtain a straight line in the oxygen recording system, assumed as steady-state photosynthesis. The irradiance calibration within the chambers was performed using a 4π quantum sensor (WALZ, Effeltrich, Germany) connected to a data logger (LI-1400, LI-COR, Lincoln, USA). Following the last light level, post-illuminatory respiration (or light respiration, R_L_) was measured in darkness. Gross photosynthetic rates were calculated by adding respiration (average between dark and light respiration) to net photosynthesis. The highest photosynthetic rate was considered as P_max_ and the photosynthetic efficiency (α) was estimated from the initial slope of the light-response curve by linear least-square regression analysis. The irradiance at the onset of saturated photosynthesis (E_k_) was obtained from the ratio P_max_/α. As photosynthetic and respiratory rates in octocorals can vary depending on the expansion or contraction of the polyps (Fabricius and Klumpp 1995), the state of the polyps was recorded during all measurements. It was found that most branches were in an intermediate state, neither with fully expanded nor contracted polyps. In the case of a different state (fully expanded, fully contracted), the measurements were repeated with a different branch to ensure comparability between measurements.

For comparison with other studies, the photosynthetic parameters were normalized by ash-free dry weight (AFDW), symbiont cell number, surface area and chlorophyll *a* concentration of the samples.

The daily integrated gross photosynthesis was calculated using photosynthetic efficiency (α) and daily variation of the irradiance at collection depth (I) as: P_t_=I_t_ x α, where P_t_ is the gross photosynthetic rate at a given time during the day (t) (see Fig. S1). The daily variation in irradiance at the collection depth during the sampling period was calculated from surface irradiance data, continuously recorded by the Oceanographic and Meteorological Academic Service (SAMMO) of the UNAM in Puerto Morelos, and the down-welling light attenuation coefficient of the reef lagoon (k_D_=0.2 m^-1^; Enríquez and Pantoja-Reyes 2005).

The photosynthesis to respiration ratio (P/R) was calculated by extrapolating the data obtained from photosynthesis-irradiance curves: (1) P_int_/R_(24h)_ = the ratio of the daily integrated gross photosynthesis, calculated as described above, assuming dark respiration over 24 h, and (2) P_int_/R_(RL:RD)_, which was calculated as described before, but here the daily respiration was extrapolated considering 12 h dark respiration (R_D_) and 12 h light respiration (R_L_). This last approach gives a more exact estimation of the P/R, as respiration during the day, when photosynthesis is active, is higher than dark respiration.

**Figure S5.** Diurnal average variation of irradiance at 2 m depth for September 2014 (black line) and the daily integrated photosynthetic rate (yellow area) calculated by using the determined photosynthetic efficiency (α) and maximum photosynthetic rate (P_max_) from measured P-E curves (H_sat_ -Hours at light saturation ≥E_k_).

The photosynthesis to respiration ratio (P/R) was calculated by extrapolating the data obtained from photosynthesis-irradiance curves: (1) P_int_/R_(24h)_ = the ratio of the daily integrated gross photosynthesis, calculated as described above, assuming dark respiration over 24 h, and (2) P_int_/R_(RL:RD)_, which was calculated as described before, but here the daily respiration was extrapolated considering 12 h dark respiration (R_D_) and 12 h light respiration (R_L_). This last approach gives a more exact estimation of the P/R, as respiration during the day, when photosynthesis is active, is higher than dark respiration.

*Surface area and volume determination*

Branches of the studied species, with the exception of *P. anceps*, *P.citrina* and *G. ventalina*, were cylindrical in shape and thus, the surface area was calculated using the equation for an open cylinder (2π ∗ radius ∗ height). In the case of *P. anceps* and *P. citrina*, which has 2 to 3 or 4-sided blade-like branches that in cross-section look like the letters ‘I’, ‘Y’ or ‘X’ (see Fig. 1), the surface area was calculated as the product of the length of the fragment, the distance between two adjacent edges (breadth), and the total number of edges present in the respective sample (either 2, 3 or 4). The area of the monoplanar species *G. ventalina* was determined by ImageJ from photographs taken of the samples.

The determinations of the surface area:volume (SA/V) ratio were performed on ten branches of each species, measuring their height and width for volume calculation and taking photographs from which their surface area was calculated as described above.

*Symbiont distribution in octocoral tissue*

Before the histological preparation the samples were rinsed in PBS (pH 7.4) for 12 h with at least three buffer changes. Branch fragments of approximately 0.5 cm length were decalcified in 10% HCl buffered solution (added with 0.7 g EDTA, 0.008 g sodium potassium tartrate tetrahydrate and 0.14 g sodium tartrate dihydrate per liter of solution). The decalcification solution was changed every day for a week and afterwards the decalcified tissue was rinsed for 2 h in Phosphate Buffered Saline and dehydrated in a gradual ethanol series (70–100%), then cleared with Xylene and embedded in paraffin (Paraplast-Leyca). Sections (8 μm thick) were obtained using a rotary microtome (Leica RM2145) and mounted on poly-L-lysine prepared microscope slides. Tissue sections were stained with Meyer`s hematoxylin and eosin procedures, and coverslipped with Organol/Limonene (Sigma O8015) mounting medium. Subsequently, photographs were obtained using an AxioImager Zeiss Microscope.

**Figure S6.** Schematic overview of the samples and subsamples used for the different experiments and analyses.

**
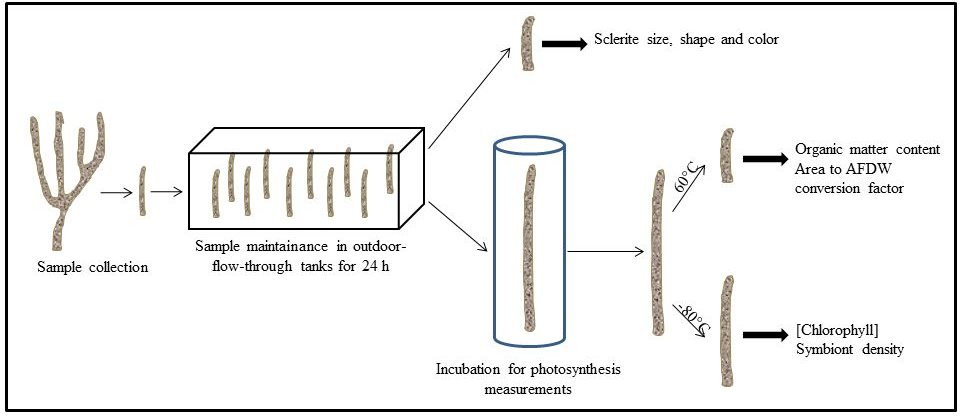
**

**Figure S7.** Examples of photos taken from sclerites of (a) *Gorgonia ventalina* and (b) *Antilllogorgia americana*.


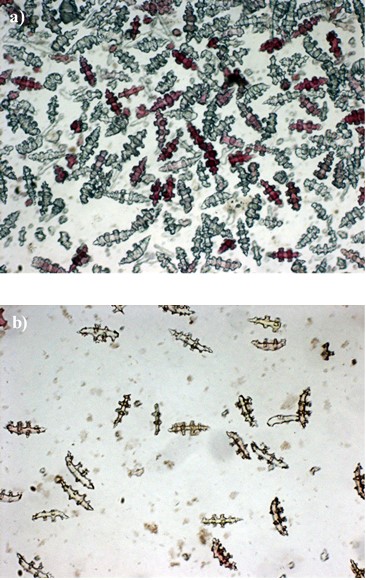


Figure S8. (A – D) Panoramic views of histological sections of *Gorgonia ventalina* and (E – H) *Plexaurella nutans*. (A and E) show sections stained with hematoxylin-eosin (HE) dyes and photographed at 4X amplification. (B and F) are the same sections observed using an eGFP fluorescence filter (Ex488 – Em509 nm), where Eosin labels the symbionts. (C and G) are merged images of HE staining overlapped with fluorescent images. The increased fluorescence signal in the area of the polyp indicates higher symbiont concentration (see black or white arrows). Boxed areas in B and F represent the sections amplified in D and H, respectively. Arrows mark symbiont cells. Scale bars are 200 μm (A–C), 500 μm (E–G) and 50 μm (D, H).

**
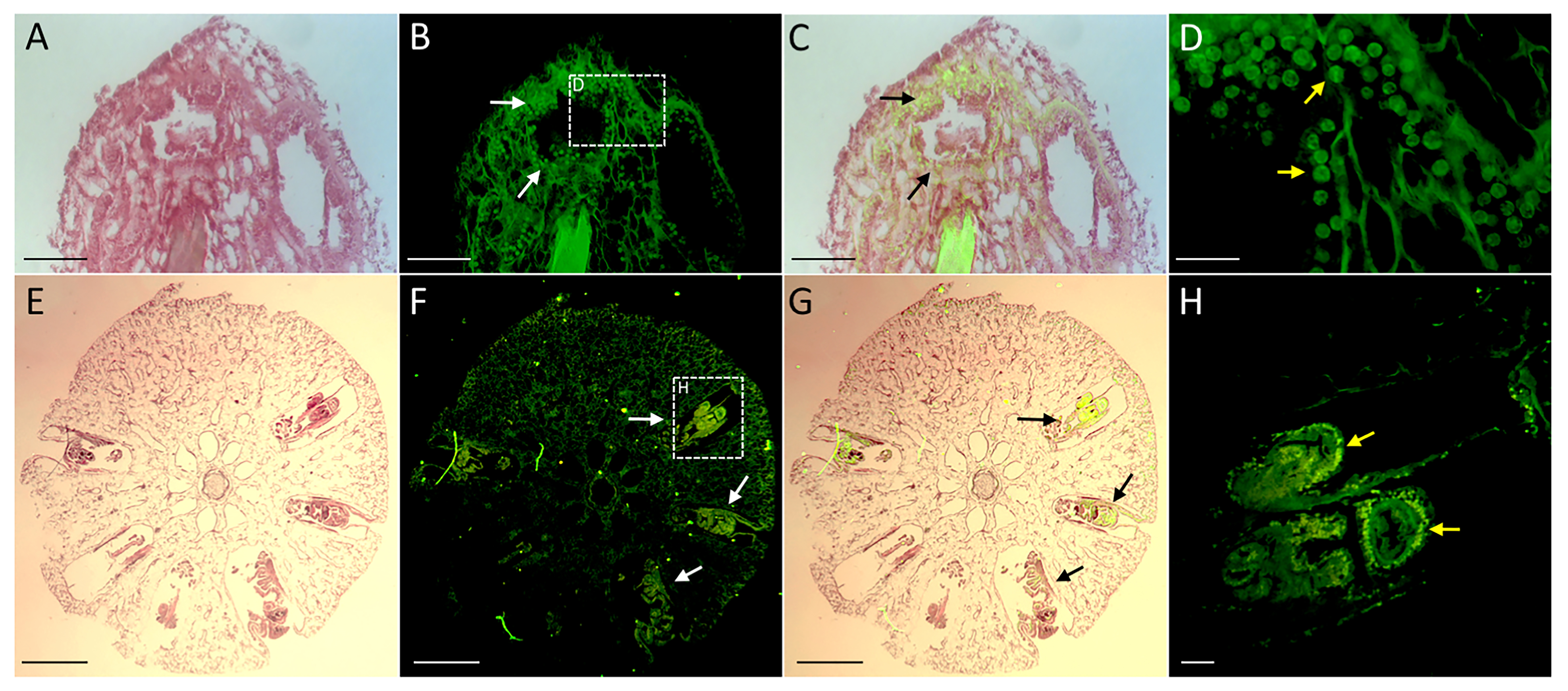
**

**Table S1**. Morphological characterization of polyps from studied Caribbean octocorals species, using data reported by Velásquez and Sánchez (2015). The polyp density was calculated based on the reported intercalyx distance. In the case of the sea whips (*P. anceps, P. citrina*), the number of polyps per cm were calculated and multiplied by two due to the bilateral arrangement of the polyps along the elevated ridges of the branches. Calyx volume and surface area were calculated using the equations for a cylinder (Volume=π ∗ calyx radius ∗ calyx depth; Surface area= 2 π ∗ calyx radius ∗ (calyx depth+radius)), as a proxy for polyp size and to calculate the SA/V of the polyp, respectively.

| Group | Species | Polyp density  (cm^-2^) | Calyx aperture  (mm) | Calyx depth  (mm) | Surface area  (mm^2^) | Calyx volume  (mm^3^) | SA/V |
| --- | --- | --- | --- | --- | --- | --- | --- |
| **Sea fan** | *Gorgonia ventalina* | 319 | 0.2 | 0.1 | 0.126 | 0.0031 | 40.0 |
|  |  |  |  |  |  |  |  |
| **Sea plumes** | *Antillogorgia bipinnata* | 126 | 0.45 | 0.36 | 0.827 | 0.0572 | 14.4 |
|  | *A. americana* | 216 | 0.45 | 0.25 | 0.671 | 0.0397 | 16.9 |
|  |  |  |  |  |  |  |  |
| **Sea whips** | *Pterogorgia anceps* | 16 | 0.88 | 0.2 | 1.768 | 0.122 | 14.5 |
|  | *P. citrina* | 14 | 1.04 | 0.29 | 2.654 | 0.246 | 10.7 |
|  |  |  |  |  |  |  |  |
| **Sea rods** | *E. mammosa* | 55 | 0.81 | 1.67 | 5.278 | 0.860 | 6.1 |
|  | *E. tourneforti* | 30 | 1.19 | 2.55 | 11.752 | 2.835 | 4.1 |
|  | *Plexaurella nutans* | 18 | 1.61 | 0.69 | 7.558 | 1.404 | 5.4 |

**Table S2.** Inorganic matter content, size, morphological traits and proportion of colored sclerites. Data represent mean ± SE. Results of one-way ANOVA are shown and significant differences between species (ANOVA, Newman-Keuls test, p<0.05) are indicated by superscript letters.

| Group | Species | Inorganic matter  (% of g DW) | Sclerite length (um) | Maximum width (um) | Minimum width (um) | Tubercles/sclerite | Colored sclerites  (% of total sclerites) |
| --- | --- | --- | --- | --- | --- | --- | --- |
|  |  |  |  |  |  |  |  |
| **Sea fan** | *Gorgonia ventalina* | 42±4^a^ | 84.3±2.3^a^ | 17.3±0.8^a^ | 12.4±0.6^a^ | 10.1±0.2^a^ | 26±4^a^ |
|  |  |  |  |  |  |  |  |
| **Sea plumes** | *Antillogorgia bipinnata* | 56±2^b^ | 97.1±1.4^a^ | 12.7±0.2^a^ | 9.1±0.2^b^ | 6.8±0.1^b^ | 18±4^b^ |
|  | *A. americana* | 43±1^a^ | 97.0±1.1^a^ | 16.0±0.2^a^ | 11.3±0.2^ab^ | 9.0±0.1^ab^ | 2±0.4^c^ |
|  |  |  |  |  |  |  |  |
| **Sea whips** | *Pterogorgia anceps* | 58±1^b^ | 152.4±2.1^b^ | 29.7±0.6^b^ | 22.3±0.5^c^ | 15.4±0.1^c^ | 20±5^bd^ |
|  | *P. citrina* | 83±0.2^c^ | 170.4±1.9^bc^ | 33.9±0.5^b^ | 25.3±0.4^d^ | 18.1±0.2^d^ | 18±2^bd^ |
|  |  |  |  |  |  |  |  |
| **Sea rods** | *Eunicea* sp. | 87±1^c^ | 184.6±8.6^c^ | 38.5±1.6^c^ | 26.3±1.0^d^ | 22.6±1.2^e^ | 1±0.2^c^ |
|  | *E. mammosa* | 73±1^de^ | 155.6±9.7^b^ | 29.6±1.9^bd^ | 18.5±1.0^e^ | 13.7±0.6^c^ | 32±5^e^ |
|  | *E. tourneforti* | 88±0.4^e^ | 124.9±11.2^d^ | 24.8±1.8^d^ | 17.0±1.0^e^ | 13.8±0.5^c^ | 0^c^ |
|  | *Plexaurella nutans* | 85±0.3^ce^ | 216.3±14.6^e^ | 55.2±4.3^e^ | 42.1±3.5^f^ | 15.0±0.7^c^ | 0^c^ |

**Table S3.** Cell numbers, pigment concentrations and chlorophyll concentration per cell (C_i_, in 10^-5^μg Chl per cell^-1^) in octocoral species with different morphological traits, normalized by ash-free dry weight (AFDW) and surface area. Data represent mean ± SE and significant differences between species (ANOVA, Newman-Keuls test, *p*<0.05) are indicated by different superscript letters.

| Parameter | **Sea fan** | | **Sea plumes** | | **Sea whips** | | **Sea rods** | | | | **One-way ANOVA** | | |
| --- | --- | --- | --- | --- | --- | --- | --- | --- | --- | --- | --- | --- | --- |
|  | *G.ventalina* | *A.bipinnata* | | *A.americana* | *P. anceps* | *P. citrina* | *Eunicea* sp. | *E.mammosa* | *E.tourneforti* | *P. nutans* | MS | F | *p* |
| **per g AFDW^-1^** |  |  | |  |  |  |  |  |  |  |  |  |  |
| Cells(*10^6^) | 131±9^ab^ | 261±61^c^ | | 133±22^ab^ | 200±32^ac^ | 213±38^ac^ | 391±63^d^ | 101±15^ab^ | 176±34^ac^ | 49±7^b^ | 53077 | 11.2 | **<0.00001** |
| Chl *a* (mg) | 0.59±0.1^a^ | 1.27±0.5^a^ | | 1.57±0.1^ab^ | 2.26±0.2^bc^ | 2.65±0.6^c^ | 1.59±0.2^ab^ | 0.56±0.1^a^ | 1.27±0.1^a^ | 0.65±0.1^a^ | 3.3 | 9.6 | **<0.00001** |
| Chl *c*_2_ (mg) | 0.15±0.02^a^ | 0.44±0.1^ab^ | | 0.45±0.03^ab^ | 1.79±0.1^c^ | 0.84±0.2^b^ | 0.56±0.1^ab^ | 0.2±0.04^a^ | 0.82±0.1^b^ | 0.63±0.1^b^ | 1.4 | 24.5 | **<0.00001** |
| Chl *a*+*c*_2_ (mg) | 0.73±0.1^a^ | 1.3±0.5^a^ | | 2.12±0.1^a^ | 4.05±0.3^b^ | 3.49±0.7^b^ | 2.15±0.4^a^ | 0.76±0.2^a^ | 2.08±0.3^a^ | 1.28±0.1^a^ | 7.8 | 13.2 | **<0.00001** |
| **per cm^-2^** |  |  | |  |  |  |  |  |  |  |  |  |  |
| Cells(*10^6^) | 0.96±0.1^ab^ | 1.1±0.1^a^ | | 0.6±0.07^a^ | 1.6±0.3^b^ | 0.9±0.2^ab^ | 2.7±0.3^c^ | 2.4±0.3^c^ | 1.0±0.1^a^ | 1.0±0.1^a^ | 3.14 | 16.65 | **<0.00001** |
| Chl *a* (μg) | 4.1±0.4^a^ | 4.9±1.8^a^ | | 7.3±0.7^ab^ | 18.3±1.8^c^ | 9.1±1.7^abd^ | 11.7±1.9^bd^ | 12.7±2.2^bd^ | 7.6±0.6^ab^ | 14.0±1.3^d^ | 119.49 | 10.98 | **<0.00001** |
| Chl *c*_2_ (μg) | 1.1±0.1^a^ | 3.9±1.0^b^ | | 2.1±0.2^ab^ | 14.5±0.9^c^ | 2.9±0.6^ab^ | 3.9±0.7^b^ | 4.7±0.7^b^ | 4.9±0.7^b^ | 13.2±1.2^c^ | 136.9 | 49.75 | **<0.00001** |
| Chl *a*+*c*_2_ (μg) | 5.2±0.4^a^ | 8.9±2.2^ac^ | | 9.4±0.8^ac^ | 32.7±2.6^b^ | 11.9±2.3^acd^ | 15.7±2.5^cd^ | 17.4±2.9^d^ | 12.5±1.3^acd^ | 27.2±2.4^e^ | 472.5 | 22.19 | **<0.00001** |
|  |  |  | |  |  |  |  |  |  |  |  |  |  |
| Chl *c*/Chl*a* | 0.27±0.04^a^ | 0.97±0.24^b^ | | 0.29±0.01^a^ | 0.81±0.05^b^ | 0.32±0.02^a^ | 0.34±0.05^a^ | 0.38±0.03^a^ | 0.64±0.05^a^ | 0.95±0.05^c^ | 0.47 | 23.37 | **<0.00001** |
| C_i_ (Chl*a*^-1^) | 0.45±0.06^a^ | 0.52±0.11^a^ | | 1.3±0.2^bc^ | 1.2±0.1^bc^ | 1.0±0.1^bd^ | 0.44±0.05^a^ | 0.5±0.06^a^ | 0.77±0.1^ad^ | 1.5±0.2^c^ | 1.00 | 12.73 | **<0.00001** |
| C_i_ (Chl*a*+*c*_2_^-1^) | 0.56±0.06^a^ | 0.83±0.1^ab^ | | 1.71±0.3^cd^ | 2.16±0.2^d^ | 1.36±0.1^bc^ | 0.59±0.06^a^ | 0.73±0.07^ab^ | 1.26±0.2^bc^ | 2.9±0.4^e^ | 3.77 | 20.89 | **<0.00001** |

**Table S4.** Photosynthetic parameters in different octocoral species. Data represent mean ± SE and significant differences between species (ANOVA, Newman-Keuls test, *p*<0.05) are indicated by different superscript letters. Maximum gross photosynthesis (P_max_), dark respiration (R_D_) and post-illuminatory respiration (R_L_) in μmol O_2_ h^-1^, α-photosynthetic efficiency in μmol O_2_ h^-1^ [μmol quanta m^-2^ s^-1^]^-1^.

| Parameter | **Sea fan** | **Sea plumes** | | **Sea whips** | | **Sea rods** | | | | **One-way ANOVA** | | |
| --- | --- | --- | --- | --- | --- | --- | --- | --- | --- | --- | --- | --- |
|  | *G. ventalina* | *A. bipinnata* | *A. americana* | *P. anceps* | *P. citrina* | *Eunicea* sp. | *E. mammosa* | *E. tourneforti* | *P. nutans* | MS | F | *p* |
| **per cm^-2^** |  |  |  |  |  |  |  |  |  |  |  |  |
| P_max_ | 2.1±0.2^abe^ | 1.8±0.1^abc^ | 1.5±0.1^acd^ | 2.6±0.1^ef^ | 1.6±0.1^acd^ | 2.3±0.3^be^ | 3.0±0.4^f^ | 0.98±0.06^d^ | 1.3±0.2^cd^ | 2.29 | 15.1 | **<0.0001** |
| α | 0.0037±0.0004^a^ | 0.0023±0.0003^bc^ | 0.0027±0.0002^ab^ | 0.0037±0.0004^a^ | 0.0024±0.0002^bc^ | 0.0034±0.0002^a^ | 0.005±0.0006^d^ | 0.0016±0.0001^c^ | 0.0020±0.0003^bc^ | <0.0001 | 13.7 | **<0.0001** |
| R_D_ | 0.45±0.03^a^ | 0.34±0.06^b^ | 0.31±0.02^b^ | 0.49±0.02^a^ | 0.24±0.02^b^ | 0.45±0.05^a^ | 0.52±0.06^a^ | 0.26±0.03^b^ | 0.23±0.02^b^ | 0.07 | 13.8 | **<0.0001** |
| R_L_ | 0.62±0.06^a^ | 0.50±0.05^b^ | 0.47±0.03^b^ | 0.69±0.03^a^ | 0.37±0.01^bd^ | 0.65±0.05^a^ | 0.90±0.1^c^ | 0.35±0.03^bd^ | 0.30±0.02^d^ | 0.21 | 23.8 | **<0.0001** |
| **per mg Chl *a*^-1^** |  |  |  |  |  |  |  |  |  |  |  |  |
| P_max_ | 442±51^a^ | 578±155^a^ | 208±16^b^ | 144±10^b^ | 145±17^b^ | 156±42^b^ | 270±53^b^ | 133±14^b^ | 113±11^b^ | 131523 | 9.569 | **<0.0001** |
| α | 0.77±0.11^a^ | 0.7±0.2^a^ | 0.4±0.03^bc^ | 0.21±0.02^b^ | 0.21±0.02^b^ | 0.24±0.04^b^ | 0.46±0.04^c^ | 0.22±0.02^b^ | 0.17±0.02^b^ | 0.26 | 16.7 | **<0.0001** |

**Table S5.** Photosynthetic and respiration rates reported in octocorals, normalized to different parameters (*7.5 h incubation without stirring).

| Species | Gross Photosynthesis (μmol O_2_) | | | | Respiration (μmol O_2_) | | Reference |
| --- | --- | --- | --- | --- | --- | --- | --- |
|  | g AFDW^-1^ h^-1^ | cm^-2^ h^-1^ | mg Chl*a*^-1^ h^-1^ | 10^-6^ cells h^-1^ | g AFDW^-1^ h^-1^ | cm^-2^ h^-1^ |  |
| **Caribbean** |  |  |  |  |  |  |  |
| *Gorgonia ventalina* |  |  |  |  | 47.5 |  | Lewis and Post (1982) |
|  | 83.8* |  |  |  | 46.9* |  | Baker et al. (2015) |
|  | **301** | **2.1** | **442** | **2.4** | **64** | **0.45** | **This study** |
| *Muriceopsis flavida* |  |  |  |  | 46.9 |  | Lewis and Post (1982) |
| *Pterogorgia anceps* |  | 2.3 | 1004 | 1.83 |  | 0.8 | Ramsby et al. (2014) |
|  | 33.7* |  |  |  | 40.6* |  | Baker et al. (2015) |
|  | **306** | **2.6** | **144** | **1.5** | **61** | **0.49** | **This study** |
| *P. citrina* | **77** | **1.6** | **145** | **1.5** | **11** | **0.24** | **This study** |
| *Antillogorgia acerosa* | 202.5* |  |  |  | 126.9* |  | Baker et al. (2015) |
| *A. bipinnata* | **210** | **1.8** | **578** | **2.1** | **39** | **0.34** | **This study** |
| *A. americana* | **182** | **1.5** | **208** | **2.9** | **38** | **0.31** | **This study** |
| *Plexaura homomalla* | 54.4* |  |  |  | 49.4* |  | Baker et al. (2015) |
| *Pseudoplexaura porosa* |  | 4.3 | 320 | 0.83 |  | 1.2 | Ramsby et al. (2014) |
| *P. wagenaari* |  | 3.3 | 150 | 0.4 |  | 1.1 | Ramsby et al. (2014) |
| *Eunicea tourneforti* |  | 1.7 | 710 | 0.53 |  | 0.51 | Ramsby et al. (2014) |
|  |  |  |  |  | 18.74 |  | Lewis and Post (1982) |
|  | **28** | **0.98** | **133** | **1.1** | **7** | **0.26** | **This study** |
| *E. flexuosa* |  |  |  |  | 19.4 |  | Lewis and Post (1982) |
|  | 15.7* |  |  |  | 14.4* |  | Baker et al. (2015) |
| *E. mammosa* | 35.0* |  |  |  | 25.0* |  | Baker et al. (2015) |
|  | **35** | **3** | **270** | **1** | **5** | **0.52** | **This study** |
| *E. calyculata* | 10.6* |  |  |  | 10.0* |  | Baker et al. (2015) |
| *E. succinea* | 31.9* |  |  |  | 28.8* |  | Baker et al. (2015) |
| *Plexaurella nutans* | 11.3* |  |  |  | 9.4* |  | Baker et al. (2015) |
|  | **68** | **1.3** | **113** | **1.4** | **11** | **0.23** | **This study** |
| *P. fusifera* | 3.8* |  |  |  | 6.3* |  | Baker et al. (2015) |
| *Briareum asbestinum* |  |  |  |  | 9.4 |  | Lewis and Post (1982) |
|  | 45.7* |  |  |  | 48.8* |  | Baker et al. (2015) |
| **Great Barrier Reef** |  |  |  |  |  |  |  |
| *Lobophytum* spp. | 34-38 |  |  |  | 12-15 |  | Fabricius and Klumpp (1995) |
| *Sarcophyton* sp. | 34-282 |  |  |  | 16-55 |  | Fabricius and Klumpp (1995) |
| *Sinularia* sp. | 37-83 |  |  |  | 17-34 |  | Fabricius and Klumpp (1995) |
| *Xenia* sp. | 74-172 |  |  |  | 19-39 |  | Fabricius and Klumpp (1995) |
| *Efflatounaria* sp. | 71-126 |  |  |  | 27-38 |  | Fabricius and Klumpp (1995) |
| *Paralemnalia clavata* | 77-88 |  |  |  | 34-36 |  | Fabricius and Klumpp (1995) |
| *P. digitiformis* | 73-109 |  |  |  | 28-38 |  | Fabricius and Klumpp (1995) |
| *Capnella lacertiliensis* | 47-96 |  |  |  | 14-31 |  | Fabricius and Klumpp (1995) |
| *Nephthea* sp. | 88-105 |  |  |  | 35-39 |  | Fabricius and Klumpp (1995) |
| *Asterospicularia* sp. | 49 |  |  |  | 22 |  | Fabricius and Klumpp (1995) |
| *Briareum stechei* | 38-77 |  |  |  | 13-39 |  | Fabricius and Klumpp (1995) |
| **Red Sea** |  |  |  |  |  |  |  |
| *Sarcophyton* sp. |  | 0.52-0.9 |  |  |  | 0.23-0.43 | Bednarz et al. (2015) |
| *Nephthea* sp. |  | 0.8-1.24 |  |  |  | 0.13-0.23 | Bednarz et al. (2015) |
| *Xenia* sp. |  | 0.14 |  |  |  | 0.06 | Bednarz et al. (2012) |
| *Heteroxenia fuscescens* |  | 0.98-2.54 |  |  |  |  | Kremien et al. (2013) |
| *Briareum asbestinum* |  |  |  |  | 9.4 |  | Lewis and Post (1982) |
| **Mediterranean** |  |  |  |  |  |  |  |
| *Eunicella singularis* |  | 1.03 |  |  |  | 0.55 | Ezzat et al. (2013) |
|  |  | 0.36 |  |  |  | 0.12-0.17 | Ferrier-Pages et al. (2015) |
| **Sydney, Australia** |  |  |  |  |  |  |  |
| *Capnella gaboensis* |  |  | 7.7-12.4 |  |  |  | Farrant et al. (1987a, b) |

References

Baker DM, Freeman CJ, Knowlton N, Thacker RW, Kim K, Fogel ML (2015). Productivity links morphology, symbiont specificity, and bleaching in the evolution of Caribbean octocoral symbioses. The ISME Journal 9:2620-2629.

Bednarz VN, Naumann MS, Niggl W, Wild C (2012). Inorganic nutrient availability affects organic matter fluxes and metabolic activity in the soft coral genus *Xenia.* J Exp Biol 215:3672-3679.

Bednarz VN, Cardini U, van Hoytema N, Al-Rshaidat MMD, Wild C (2015). Seasonal variation in dinitrogen fixation and oxygen fluxes associated with two dominant zooxanthellate soft corals from the northern Red Sea. Mar Ecol Prog Ser 519:141-152.

Enríquez S, Pantoja-Reyes NI (2005). Form-function analysis of the effect of canopy morphology on leaf self-shading in the seagrass *Thalassia testudinum.* Oecologia 145:235-243.

Ezzat L, Merle P-L, Furla P, Buttler A, Ferrier-Pagès C (2013). The response of the Mediterranean gorgonian Eunicella singularis to thermal stress is independent of its nutritional regime. PLoS ONE 8:e64370.

Fabricius KE, Klumpp (1995). Widespread mixotrophy in reef-inhabiting soft corals: the influence of depth, and colony expansion and contraction on photosynthesis. Mar Ecol Prog Ser 125:195-204.

Farrant PA, Borowitzka MA, Hinde R, King RJ (1987a). Nutrition of the temperate Australian soft coral *Capnella gaboensis*. I. Photosynthesis and carbon fixation. Mar Biol 95:565-574.

Farrant PA, Borowitzka MA, Hinde R, King RJ (1987b). Nutrition of the temperate Australian soft coral *Capnella gaboensis*. II. The role of zooxanthellae and feeding. Mar Biol 95:575-581.

Ferrier-Pagès C, Reynaud S, Béraud E, Rottier C, Menu D, Duong G, Gévaert F (2015). Photophysiology and daily primary production of a temperate symbiotic gorgonian. Photosynth Res 123:95-104.

Kremien M, Shavit U, Mass T, Genin A (2013). Benefit of pulsation in soft corals. Proc Natl Acad Sci 110:8978-8983.

Lewis JB, Post EE (1982) Respiration and energetics in West Indian Gorgonacea (Anthozoa, Octocorallia). Comp Biochem Physiol A Comp Physiol 71: 457–459.

Ramsby BD, Shirur KP, Iglesias-Prieto R, Goulet TL (2014). *Symbiodinium* photosynthesis in Caribbean octocorals. PLoS ONE 9:e106419.
